# Genetic Diversity of Porcine Circovirus 2 in Wild Boar and Domestic Pigs in Ukraine

**DOI:** 10.3390/v14050924

**Published:** 2022-04-28

**Authors:** Nataliia Rudova, Jeremy Buttler, Ganna Kovalenko, Mykola Sushko, Vitaliy Bolotin, Larysa Muzykina, Oleksandr Zinenko, Borys Stegniy, Yurii Dunaiev, Mykola Sytiuk, Anton Gerilovych, Devin M. Drown, Eric Bortz, Oleksii Solodiankin

**Affiliations:** 1 National Scientific Center Institute of Experimental and Clinical Veterinary Medicine, 61023 Kharkiv, Ukraine; rudovanatawa@ukr.net (N.R.); vbolotin@hotmail.de (V.B.); boris.stegniy@gmail.com (B.S.); dunaev2975@gmail.com (Y.D.); antger2011@gmail.com (A.G.); 2 Department of Biology & Wildlife, University of Alaska Fairbanks, Fairbanks, AK 99775, USA; jdbuttler@alaska.edu; 3 Institute for Veterinary Medicine, 03151 Kyiv, Ukraine; ak2388@cam.ac.uk (G.K.); loramuzykina@i.ua (L.M.); snp1978@ukr.net (M.S.); 4 Department of Biological Sciences, University of Alaska Anchorage, Anchorage, AK 99508, USA; 5 Division of Virology, Department of Pathology, University of Cambridge, Cambridge CB2 0QN, UK; 6 State Scientific and Research Institute of Laboratory Diagnostics and Veterinary and Sanitary Expertise, 03151 Kyiv, Ukraine; m.i.sushko@gmail.com; 7 Department of Mycology and Plant Resistance, The Museum of Nature, V. N. Karazin Kharkiv National University, 61000 Kharkiv, Ukraine; oleksandrzinenko@gmail.com; 8 Institute for Arctic Biology, University of Alaska Fairbanks, Fairbanks, AK 99775, USA

**Keywords:** porcine circovirus, PCV2, domestic pig, wild boar, genotype, phylogenetics, MinION, Ukraine

## Abstract

Porcine circovirus type 2 (PCV2) is responsible for a number of porcine circovirus-associated diseases (PCVAD) that can severely impact domestic pig herds. For a non-enveloped virus with a small genome (1.7 kb ssDNA), PCV2 is remarkably diverse, with eight genotypes (a–h). New genotypes of PCV2 can spread through the migration of wild boar, which are thought to infect domestic pigs and spread further through the domestic pig trade. Despite a large swine population, the diversity of PCV2 genotypes in Ukraine has been under-sampled, with few PCV2 genome sequences reported in the past decade. To gain a deeper understanding of PCV2 genotype diversity in Ukraine, samples of blood serum were collected from wild boars (*n* = 107) that were hunted in Ukraine during the November–December 2012 hunting season. We found 34/107 (31.8%) prevalence of PCV2 by diagnostic PCR. For domestic pigs, liver samples (*n* = 16) were collected from a commercial market near Kharkiv in 2019, of which 6 out of 16 (37%) samples were positive for PCV2. We sequenced the genotyping locus *ORF2*, a gene encoding the PCV2 viral capsid (Cap), for 11 wild boar and six domestic pig samples in Ukraine using an Oxford Nanopore MinION device. Of 17 samples with resolved genotypes, the PCV2 genotype b was the most common in wild boar samples (10 out of 11, 91%), while the domestic pigs were infected with genotypes b and d. We also detected genotype b/d and b/a co-infections in wild boars and domestic pigs, respectively, and for the first time in Ukraine we detected genotype f in a wild boar from Poltava. Building a maximum-likelihood phylogeny, we identified a sublineage of PCV2 genotype b infections in both wild and domestic swine, suggesting a possible epizootic cluster and an ecological interaction between wild boar and domestic pig populations in northeastern Ukraine.

## 1. Introduction

### 1.1. Epidemiology of Porcine Circovirus 2-Associated Diseases in Swine

Between 2017 and 2021, the world produced 96–112 million tons of pork per year [1]. Swine raised in commercial facilities and backyard farms are at risk for epizootic infections. Some swine diseases, such as African swine fever (ASF) can only be controlled through biosecurity measures as there is no widely distributed vaccine for the causative virus [2]. The pork industry uses vaccines to avoid the increased costs from animal deaths and weight loss caused by other pathogens, particularly highly transmissible viruses such PRRSV (arterivirus), PEDV (coronavirus), classical swine fever (flavivirus), swine influenza A virus, and porcine circovirus type 2 (PCV2) [3]. However, vaccine formulations must often be updated to maintain efficacy against circulating genotypes of swine viruses.

Pigs of all breeds, as well as wild boars, are susceptible to porcine circovirus (type) 2 infection. PCV2 can cause an array of diseases collectively known as porcine circovirus-associated diseases (PCVAD), with diverse clinical presentations [4,5]. PCVAD diseases include symptoms that cause weight loss (wasting), postweaning multisystemic wasting syndrome), interstitial pneumonia (porcine respiratory disease complex), and dermatitis (porcine dermatitis and nephropathy syndrome). PCV2 co-infections with other pathogens or even different PCV2 variants may exacerbate PCVAD [4,5,6].

Transmission of the PCV2 can occur horizontally (pig-to-pig contact) and vertically (sow to piglet). In domestic pigs, the source of infection are sick and latently infected animals of either sex, which can excrete the pathogen in feces, urine, saliva, milk, nasal, or ocular secretions [7,8]. As a non-enveloped virus, PCV2 can survive for a long time in biological fluids and feces and is easily transmitted orally or via respiratory routes to other animals, and the amount of virus passed along during transmission can vary depending on the age of the animals [8].

The pathogen is transmitted directly through contact with infected animals or through the use of virus-contaminated feed, sexual contact, or artificial insemination [8,9,10]. The attack rate of an infected pig is unknown, but two studies suggest transmission takes six weeks. In one study, PCV2 was transmitted 42 days after infection between infected and seronegative piglets in direct contact [11]. In another study, after intranasal infection of boars, PCV2 was found to be secreted in semen for 47 days, suggesting that a sexual transmission route of PCV2 also exists [6].

It is thought that PCV2 can spread between countries through the migration of wild boar, or through the trade of contaminated feed or subclinically infected pigs [8,12]. When a new genotype of PCV2 enters a country, it can transfer between domestic pigs and wild boar populations, thus wild boars may act as both a reservoir and a vector for PCV2 [13].

Whole-virus inactivated, and recombinant vaccines have reduced PCVAD incidence and increased weight gain in subclinically infected swine herds and have reduced the incidence of PCV2 infections [12]. However, vaccines have not prevented PCV2 infections, which has led to an increased selection pressure for escape variants in vaccinated pigs [9,12]. In one case, PCV2 in vaccinated pigs had the same non-synonymous nucleotide mutation rate as PCV2 in unvaccinated pigs [14]. The presence of PCV2 in vaccinated pigs, and possible emergence of escape variants, has spurred recent vaccine developments and enhanced genomic surveillance in several countries including China, Italy, Korea, and the US [13,15,16,17].

### 1.2. PCV2 Genome and Genotypes

PCV2 is a non-enveloped virus with an icosahedral (T = 1) capsid that contains a single stranded circular DNA genome of 1.7 kb [18]. The genome of PCV2 contains two major open reading frames (ORFs), and at least four other functional ORFs [18,19,20,21]. The two major genes are the replicase (*ORF1*) encoding Rep and Rep’, and capsid (*ORF2*) encoding the capsid (Cap; also, Cp) protein. Genotypes of PCV2 are determined by *ORF2*, encoding Cap, with at least 8 genotypes (a–h) recognized [22].

Ukraine is a major pork producer and harbors both large domestic swine (commercial and backyard) and wild boar populations. These swine populations have been subjected to PCV2 infection [23] and swine influenza [24] in wild boar, and porcine teschovirus 1 [25] and ASF in both wild and domestic animals [26,27]. The circulation of PCV2 genotypes in swine in Ukraine is poorly understood and suffers from the sporadic nature of surveillance. In 2015, Ukraine’s major PCV2 genotype was b, with genotypes a, d, and g also in circulation [28,29,30]. Genotype d is currently a common genotype, may replicate better in vaccinated pigs, and was rare before 2014 [5,12,31]. With just 29 sequences reported in NCBI, it is likely that PCV2 diversity in Ukraine has been underestimated.

To understand PCV2 circulation in Ukraine, we performed nanopore sequencing of PCV2 *ORF2* (Cap) amplicons, generated from archived serum samples from wild boar collected in 2012, and liver samples from domestic pigs collected in 2019 [32]. We assembled one complete PCV2 genome from a wild boar. We conducted a phylogenetics analysis of *ORF2* and found the most common PCV2 genotype in Ukraine was genotype b. We also detected genotype a in wild boar and genotype d in domestic swine. Three domestic pigs and two wild boars also had evidence of co-infection with two PCV2 genotypes.

## 2. Materials and Methods

### 2.1. Sample Collection

Blood sera were collected from hunted wild boar (*n* = 107) in the November–December 2012 hunting season in Ukraine. Samples were collected in 10 oblasts (provinces) across Ukraine (Poltava, Sumy, Zaporizhzhia, Chernihiv, Chernivetsk, Cherkasy, Kherson, Lviv, Volyn, and Luhansk). Liver samples (*n* = 16) from clinically healthy pigs were collected from a commercial market in Kharkiv oblast in 2019. PCV2 vaccination status was not available. Sera and liver samples were stored at −30 °C, and total DNA was extracted from 200 μL of serum, or 10% liver suspension in PBS, using a commercial kit (QIAamp cador Pathogen Mini Kit, Qiagen, Valencia, CA, USA) according to the manufacturer’s instruction. Extracted DNA was eluted in 100 μL elution buffer and stored at −30 °C at the NSC IECVM biosafety level 2 laboratory in Kharkiv, Ukraine.

### 2.2. Diagnostic PCR to Detect PCV2

All DNA samples were investigated for PCV2 viremia using conventional Taq PCR with a Maxima Hot Start Green PCR Master Mix (ThermoFischer Scientific, Waltham, MA, USA). Primers targeting *ORF1* were forward primer PCV-2F 5’-GAAGACGAGCGCAAGAAAATACG-3’, and reverse primer PCV-2R 5’-CCAATCACGCTTCTGCATTTTCCC-3’. The diagnostic primers flank a 421 nt variable region of the tail Rep gene [23]. For each sample, 5 μL of the eluate was run in a 25 μL reaction, with an annealing step at 60 °C, on a conventional PCR machine (Biometra TAdvanced Thermal Cycler, Analytik Jena GmbH).

### 2.3. Amplification and Sequencing of ORF2 (Cap) and PCV2 Genomes

Positive swine DNA samples were used to generate of *ORF2* DNA amplicons (798 bp) by PCR for sequencing, using primers reported previously [23] Rudova et al., 2019). Briefly, PCR for *ORF2* sequencing was performed using conventional PCR and Platinum SuperFi II Taq DNA Polymerase (ThermoFischer/Invitrogen, Waltham, MA, USA). Primers targeting *ORF2* were forward primer PCV-2seqF 5’-CCCATGCCCTGAATTTCCA-3’, and reverse primer PCV-2seqR 5’-CCAATCACGCTTCTGCATTTTCCC-3′. For each positive DNA sample, 10 μL of the eluate was run in a 50 μL reaction, with an annealing step at 55 °C, on a Biometra TAdvanced Thermal Cycler PCR machine (Analytik Jena GmbH, Jena, Germany). To amplify nearly the complete genome of PCV2 (1768 bp), a three-primer amplification scheme was employed using the same PCR machine with primers and conditions reported in the literature [33]. All PCR products were electrophoresed on a 1.5% agarose gel stained with ethidium bromide and visualized using an ultraviolet transilluminator to assess DNA quality and concentration. PCR amplicons were purified using a SPRI beads (Agencourt AMPure XP beads at 1:1 sample:beads) prior to sequencing.

### 2.4. Nanopore Sequencing and Bioinformatics

All samples were sequenced in multiplex on a nanopore MinION Mk1B device in veterinary labs in Ukraine (NSC IECVM in Kharkiv; SSRILDVSE and NAAS IVM in Kyiv), using an end-ligation (SQK-LSK109) with a native barcoding (EXP-NBD104) protocol according to the manufacturer’s instructions (ONT: Oxford Nanopore Technologies, Oxford, UK). We basecalled and demultiplexed the raw sequence reads using Guppy version 3.6 (ONT). To generate consensus sequences from the raw reads, and discover mixed genotype co-infections, we took a competitive reference-based assembly approach. We filtered, binned, and competitively mapped ORF2 reads to a subset of 66 PCV2 ORF2 sequences, with at least one known references from genotypes a, b, d, f, and g ORF2 sequences, with minimap2 v2.22 [34] to generate consensus genomes. For additional detailed description of bioinformatics methods for consensus genome assembly, see Supplementary Materials.

### 2.5. Sequence Curation and Availability

We found references for manual curation by blasting each consensus against the PCV2 database available in Genbank (taxid: 85708). The top hit for each consensus was then used to detect and remove indels in the consensus. We also checked and corrected reading frames using transeq from the emboss package v6.6 [35]. All sequences were deposited in NCBI GenBank under BioProject (PRJNA832222) and associated Accessions.

### 2.6. Phylogenetics Analysis

We built a database for phylogenetic tree construction with the consensus genomes from our samples, and with PCV2 ORF2 sequences on GenBank. Our database contained 5862 ORF2 sequences downloaded from Genbank (Supplementary File S1). ORF2 sequences in our database were aligned with Mafft V7.407 [36] and manually inspected to remove sequences with early stop codons or incomplete reading frames with Genious v2020.2.1 [37]. We removed recombinant sequences from the inspected ORF2 sequences with RDP4 v4.101 using similar settings to those described previously [18]. We then removed all gaps and stop codons in our remaining, aligned ORF2 sequences, using Geneious v2020.2.1. Most of the ORF2 sequences in our database were detected as recombinant, leaving us with 429 ORF2 sequences in our final database (Supplementary Material Table S1).

We inferred a maximum-likelihood (ML) tree using IQtree, our consensuses, and a sub-sample of our ORF2 sequence database. Sub-sampling was conducted with cd-hit using parameter -c 0.985 to reduce our database to a manageable size. After sub-sampling, the ORF2 sequences left in our database were at least 1.5% different. We added ORF2 sequences from Ukraine that were removed by sub-sampling back into our sub-sampled database. We built a consensus tree with IQtree v1.6.12 [38] using parameters -st CODON -m MFP+MERGE -bb 1000, the sub-sampled ORF2 database, and the ORF2 genes from our consensuses. The tree inference was statistically tested (1000 ultrafast bootstraps). The consensus tree was edited in R studio using the treeio, ape, ggplot2, and ggtree [15,38].

### 2.7. Mantel Test

Studies of the correlation of geographical and genetic distances of Ukrainian isolates were completed using the Mantel test [39]. We analyzed the Mantel test output using R software (https://www.r-project.org, accessed on 4 February 2022) as described in [40].

## 3. Results

### 3.1. Detection of PCV2 in Wild Boar and Domestic Pigs in Ukraine

**Figure 1:**
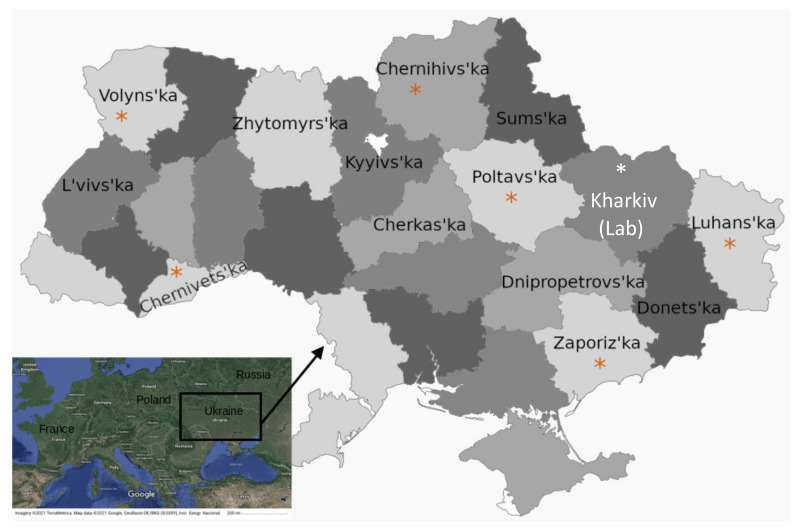
Sampling for PCV2 in Ukraine in wild boar and domestic pigs. Map of Ukraine showing the oblasts (provinces) where wild boar blood serum samples were collected opportunistically over the November–December 2012 winter hunting season (red asterisks). Domestic pig liver samples were collected from a commercial market near Karhkiv in 2019, at the site of the NSC IECVM veterinary lab (white asterisk). Other PCV2 sequences downloaded from GenBank were from the named oblasts on the map.

To analyze PCV2 infection in wild boar, blood sera (*n* = 107) were opportunistically collected from disease surveillance of hunted wild boar over the November–December 2012 winter hunting season in Ukraine. Thirty-four (31.8%) samples were positive for PCV2, and samples of good DNA quality from 6 different regions across Ukraine (Zaporizhia, Chernivtsi Poltava, Volyn, Chernihiv, and Luhansk) were selected for sequencing (Table 1 and Figure 1).

To genotype PCV2 infection in domestic swine, liver samples (*n* = 16) were collected in a commercial market near Kharkiv in 2019 (Figure 1). Although PCV2 infection and PCV2 vaccination status was not known, pork products sold at markets in the Kharkiv region appeared to be derived from generally healthy animals. Nevertheless, in this small sampling, 6 of 16 liver samples (37%) were positive for PCV2 by PCR and ORF2 gene sequenced.

### 3.2. Subtyping of PCV2 by Sequencing

To analyze the genotype(s) of PCV2 in wild boar and domestic pig samples, we amplified and sequenced a 798 bp amplicon of ORF2 (Cp) using a nanopore (MinION) platform. We generated consensus sequences from nanopore sequence reads by polishing our best *ORF2* read in each sample with Medaka, and tested for co-infections (mixed genotype infections), with the steps mentioned in our supplemental methods. Co-infections and genotypes were confirmed using medaka_variant, with a reference from genotypes a, b, c, both d clades, f, g, and h (Table S2). All samples, except one (Luhansk-1), had a read depth of 14,941–635,035 reads, had at least 99% of reads from the major and minor variant, and a mean Q-score of 12.9–14.6 for reads matching the major PCV2 genotype identified (Table 2 and Table S2).

### 3.3. PCV2 Full-Length Genome from Ukraine

We attempted to generate full length genomes of 10 total DNA samples using a 3-primer PCR protocol that was reported previously [33]. Although all samples yielded long reads, we successfully assembled one complete genome from a wild boar (3194 reads with mean Q = 12.5, read depth of 32× for reads >1700 nt length). This was a genotype **b** genome annotated as PCV2/Chernihiv-1/2012 (1767 nt length; Accession: pending). This genome matched its closest relative in NCBI GenBank for 1764/1767 nt with 3 single nucleotide variations (SNV). The amino acid sequences of the Rep, ORF2 (Capsid), and ORF3 proteins of this virus are an exact match to its closest PCV2 genotype **b** relatives that were sequenced in the same year (2012) in China (JX406426.1 and HQ395035.1).

### 3.4. Co-Infections with Two PCV2 Genotypes

We tested for co-infections in our samples with a co-infection pipeline that quantitatively mapped reads to different PCV2 genotypes (see Table S2). We found five incidences of co-infection by more than one PCV2 genotype (Table 3). Two of twelve wild boar samples that were positive for PCV2, both collected in Chernivtsi in 2012, had >3% of their reads from a co-infection with another (minor) PCV2 genotype, **a**, while three of six infected domestic pig samples collected in 2019 had minor variants contributing 5–28% of their reads from a co-infection with genotypes b and d. Thirteen of eighteen resolved isolates (60%) showed evidence of only one (major) genotype of PCV2, according to our sequencing analysis (Table 2).

### 3.5. Phylogenetic Analysis of PCV2 in Ukraine

**Figure 2:**
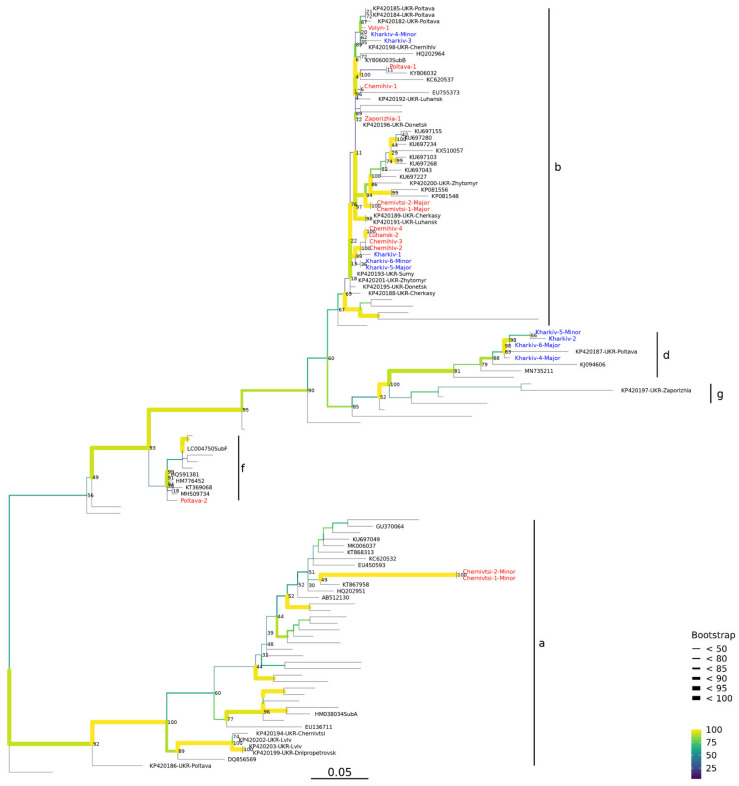
Phylogeny of porcine circovirus 2 in Ukraine. A maximum-likelihood tree was inferred for PCV2 ORF2 (Cap) genes from our study (red, wild boar; blue, domestic pig), and sequences downloaded from GenBank (Accessions). PCV2 co-infections sequenced in this study are labeled (–major and –minor), with PCV2 subtype clades identified (a, b, d, g, and f). The genotype-defining reference sequences are the major PCV2 lineage assignment with indicated GenBank Accessions (designated sub or described in the text).

We analyzed the relationship among PCV2 isolates and genotypes identified in Ukraine through phylogenetic tree construction. PCV2 ORF2 (Cap) amplicon sequences were aligned using MAFFT V7.407 with a set of 97 contemporary PCV2 reference sequences, including all previous good quality sequences from Ukraine, sub-sampled from NCBI GenBank (Supplementary File S1). The alignment was used for inference of a maximum-likelihood (ML) tree of PCV2 Cap sequences using IQtree v1.6.12. We found that the PCV2 sequences from Ukraine grouped into clades containing reference genomes representing genotypes a, b, d, f, or g. Bootstrap support analysis allowed inference of potential PCV2 transmission chains or clusters within genotypes **b**, **d**, and **a** through analysis of the phylogenetic tree (Figure 2).

Most of the major PCV2 strains from wild boar across Ukraine (10 of 11 resolved, BB = 93 of 100 bootstraps) fell into the genotype **b** clade, along with a reference genome from Ukraine (KP420197) isolated from a wild boar in Zaporizhzhia in 2010 (Figure 2). In addition, two of the minor co-infecting sequences in wild boar grouped with genotype **b**. The remaining wild boar PCV2 isolates included minor genotype **a**, detected as two co-infections of genotype **b** (Chernivitsi-1 and -2). These two minor variants fell into a genotype **a** (reference sequence HM038034) that contained PCV2 strains of genotype **a** from wild boar in Ukraine in 2012 (KP420202, KP420203, KP420186, KP420194, and KP420199). A divergent PCV2 genotype **f** sequence was also found in wild boar (Polatava-2, BB = 93) in a clade with a 2013 genotype **f** reference strain (LC004750). Genotype **f** has been found in Asia and elsewhere, but it has not previously been found in Ukraine.

In domestic swine samples collected from Kharkiv in 2019, 3 of 6 were PCV2 genotype **b**, and 3 of 6 were inferred as genotype **d** (BB = 60), with a reference PCV2 genotype **d** genome from China in 2012 (KC515014), and not with genotype **g** (BB separating clades with genotype d and g = 100). The Ukraine domestic pig genotype **d** sequences fell into a sublineage (BB = 91) with a Ukraine isolate from a wild boar in Poltava in 2012 (KP420187). One domestic pig infected by PCV2 genotype **b** also had a minor genotype **d** co-infection (Kharkiv-5).

### 3.6. Co-Circulation of PCV2 Genotypes

These results suggest there was co-circulation of at least three PCV2 genotypes (**b**, **a**, and **f**) in wild boar in 2012, and two genotypes (**b** and **d**) in domestic pigs in 2019, in Ukraine (Table 2). Co-infections between genotypes **b** and **a** in wild boar, and **b** and **d** in domestic pigs, were also observed (Table 3), and are not unprecedented considering the finding of animals in Ukraine infected solely with these genotypes. Analysis of the introduction and epizootic spread of PCV2 genotypes among wild boar, and the infection of domestic swine, is considered in the Discussion.

### 3.7. Genetic Structure of PCV2 Genotype b

**Figure 3:**
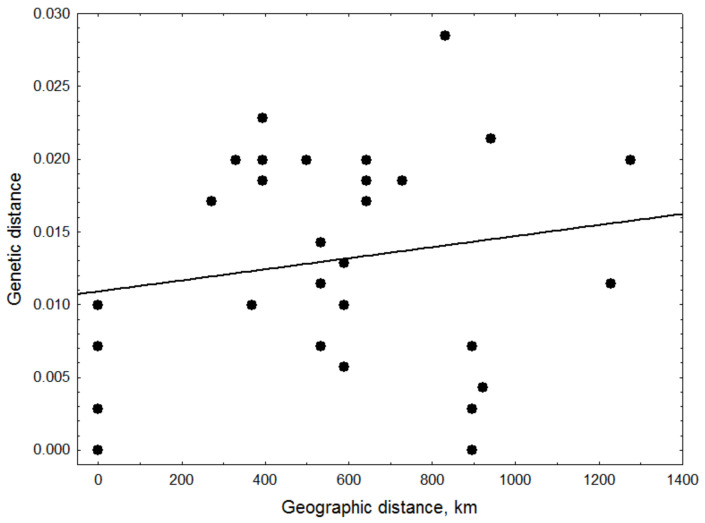
Correlation of geographical and genetic distances for PCV2 genotype b. Eight genotype b wild boar ORF2 (Cap) sequences and genetic distances (*y*-axis) were correlated with distance between the geographical centers of their respective locations (*oblasts*) in Ukraine (*x*-axis), and analyzed using the Mantel test, with significance tested by linear regression (*p*-values, *not significant*).

We analyzed the genetic structure of the PCV2 genotype **b** population circulating among wild boar populations in Ukraine in 2012 by estimating the correlation of geographical and genetic distance using the Mantel test. We excluded repetitive sequences Chernivtsi-1 and Chernivtsi-2, and the genotype **f** Poltava-2, and the domestic swine samples. The remaining genotype **b** wild boar sequences (Table 2) formed eight conditional genetic units associated with their pairwise geographic distances in Ukraine. We found no statistically significant relationship between geographical distance and the genetic structure of wild boar genotype **b** sequences (Mantel test *p* = 0.763; correlation *p* = 0.3742; Figure 3). Thus, we did not detect a geographical structure for the population of wild boars carrying this genotype of PCV2 in the limited data set available.

## 4. Discussion

Despite a small (1.7 kb) ssDNA genome with limited coding capacity (a replicase, a capsid protein, and accessory proteins), porcine circoviruses are widely spread pathogens that cause a diverse spectrum of diseases (PCVAD) in swine [3,4,5]. We conducted a genomics inquiry to identify the genotypes of PCV2 that have spread through the large wild boar and domestic pig populations of Ukraine. The PCV2 genotypes **a**, **b**, **d**, and **g** have been detected previously, if sporadically, in Ukraine, mainly in wild boar [30], with additional sequences deposited in GenBank (from an unpublished study by Podgorska, K., Sytiuk, M., Stepniewska, K. & Kus, K. (2015). Porcine circovirus type 2 infections in wild boars in Ukraine. NCBI GenBank, accessed 27 April 2022, available at: https://www.ncbi.nlm.nih.gov/sites/myncbi/eric.bortz.3/collections/61811344/public/) (Table S3). We applied a PCR diagnostics assay that we had previously developed [23] to examine archived blood samples from wild boar that had been opportunistically hunted across Ukraine in 2012, and found a PCV2 prevalence of 31.8% (*n* = 34 of 107 samples). We also collected and identified PCV2 in a sampling of domestic pork products (liver) from a market in Kharkiv in 2019 (37% prevalence).

### 4.1. PCV2 Genotypes Detected in Ukraine

To understand PCV2 diversity and spread, we sequenced ORF2 (capsid, Cap) genes in 18 samples, and recovered 17 sequences for PCV2 subtyping and phylogenetics analysis. We found that both domestic pigs and wild boars were infected with PCV2 genotypes **b** and **d**, with genotype **b** being the most common genotype with 10 of 12 wild boar and 5 of 6 domestic pig samples (83% genotype **b**). We found evidence of PCV2 co-infections of genotypes (major/minor) **b**/**d**, **d**/**b**, and **b**/**a**. In 5 of 18 cases (28%), there is a dominant (major) genotype and co-infection with a different second (minor) genotype. This finding is similar to reports of PCV2 co-infections in previous studies, and **b**/**d** co-infections were the most common co-infection seen in the US in 2015 [31]. We also found one wild boar sample infected with PCV2 genotype **f**, a genotype not previously detected in Ukraine. Although limited by a relatively small number of sporadic samples in our own and previous studies, cumulatively, our phylogenetics analysis suggests that the large swine population in Ukraine might host a diversity of PCV2 genotypes, riddled with wild boar-to-domestic pig transmission events (Figure 2).

### 4.2. Transmission and Epidemiology

Recent studies have investigated the prevalence and genotype diversity of PCV2 in wild boar and domestic swine in the forested countries of Central and Eastern Europe. PCR studies indicate that the prevalence of PCV2 is typically 20–40% in domestic pigs on farms in regions of PCV2 prevalence, even with vaccination. For example, PCV2 was found in 25% of pigs in the US during 2015 [31,41]. Numerous studies have investigated PCV2 in European domestic and wild pigs [42,43]. Serological studies also indicate a high seroprevalence, for example, 48% in a study of Iberian wild pigs [44]. Apparently, PCV2 maintains a high enzootic (endemic) prevalence in swine herds in countries in Europe with similar agricultural practices, a significant wild boar population, and mixed forest/farmland ecologies, including Poland, Hungary, Germany, Romania, and other countries, including Ukraine. In wild boars, PCV2 DNA was detected in 20.5% of pathological material samples in Hungary [45], 18.1% in Germany [46], 13.5% in Romania [47], and up to 75% in Poland [48], illustrating a wide variation that may be due to outbreak dynamics or host susceptibility.

PCV2 genotypes in wild boar have been detected in domestic pigs, suggesting the possible spread of PCV2 between wild boar reservoirs and domestic swine, which can contribute to the diverse spectrum of diseases in domestic pigs [8,13]. There is genetic evidence for sharing of PCV2 strains among wild boar and domestic pigs in Europe, a pattern we found in phylogenetics analysis of PCV2 genotypes in Ukraine (Figure 2). Recent studies showed PCV2 sequences from wild boar and domestic pigs were closely related in Slovenia [49] and Germany [46], as well as in Hungary and Romania [45,47]. In Poland, nine PCV2 genotype **b** sequences from wild boar showed very high identity with sequences from domestic pigs, a pattern also noted among genotype **a** [48,50].

The genetic similarity of PCV2 sequences indicates a shared ecological interaction between wild boar and domestic pigs, with sufficiently close contact for the transmission of PCV2 and other pathogens such as ASF [8,45,51]. Thus, the introduction of new antigenically distinct strains of PCV2 may lead to outbreaks (epizootics) in swine and symptomatic cases of PCVAD.

However, with its high global prevalence, closely related genetic isolates of PCV2 genotype **b** have also been found far afield. For example a wild boar isolate from Germany was closely related to a domestic pig isolate from China [46]. In Hungary, some strains were closely related to the isolates in Canada, while others were similar to the isolates from Germany. The full genome we sequenced, PCV2/Chernihiv-1/2012 from a wild boar, was very similar (3 nt differences) to contemporary strains from China. This confounding data suggests that PCV2 may also benefit from anthropogenic modes of transmission, possibly involving transboundary trade in live pigs and/or pork products.

### 4.3. A Wild Boar Transmission Chain

We detected a potential transmission chain between wild boars in the non-neighboring *oblasts* (provinces) of Luhansk and Chernihiv in Ukraine. At least two other potential transmission chains between non-neighboring *oblasts* have also been found [30], suggesting that the circulation of PCV2 is under-sampled in Ukraine. It is possible that a combination of wild boar migrations and human activity, such as live pig trade and the mixing of herds from different farms, may contribute to PCV2 spread [5,8]. In addition, PCV2 co-infections (mixed infections with 2 or more genotypes) and the possibility of genomic recombination events, may contribute to the epidemic and genetic diversity of PCV2 genotypes [52].

A small cluster of PCV2 genotype **b** sequences from Luhansk (Luhansk-2) and Chernihiv (Chernihiv-2, -3 and -4) had a branch length of zero in the ML phylogenetic tree (Figure 2). This suggests the existence of an epizootic transmission chain between Luhansk and Chernihiv, or along the border region in northeastern Ukraine (BB = 100). We also found a cluster of PCV2 genotype **b** in domestic pig liver samples from a commercial market in Kharkiv *oblast*, proximal to this branch (BB = 99). Although separated by seven years, the presence of PCV2 in wild boars hunted in 2012 and in domestic pig samples from 2019 is remarkable and suggests that there is an endemic sublineage of PCV2 genotype **b** that spans the northeastern region of Ukraine. This region—which includes northern parts of Kyiv, Chernihiv, Sumy, Kharkiv, and Luhansk *oblasts* in Ukraine and spans the border with the Russian *oblasts* of Kursk, Belgorod, and Voronezh—harbors a considerable wild boar population that has also exhibited evidence of cross-border transmission of African swine fever [26,53], as well as epizootics of swine influenza [24] and porcine teschovirus-1 [25].

Other potential PCV2 transmission chains in our dataset can be inferred among wild boar within Chernivsti in 2012 (Chernivitsi-1 and -2). These two hunted wild boar samples were collected in close proximity in November–December 2012 and share both PCV2 of the dominant major genotype **b** (BB = 100), and genotype **a** minor variant (BB = 100, approximately 3% of reads in each sample), although further details are not available. They exist within a poorly sampled sublineage or subclade of PCV2 genotype **b**, with representatives from wild boar from Zhytomyr in 2012, Cherkasy, Luhansk, and numerous isolates from elsewhere in Eurasia (BB = 76). Other members of this genotype **b** sublineage may include isolates from Poltava, Chernihiv, Luhansk, and Volyn *oblasts*, along with one of our PCV2 sequences that was from domestic pig liver in Kharkiv (Kharkiv-3).

### 4.4. PCV2 Genotypes in Ukraine Reflect Global Diversity

By phylogenetic analysis, we found examples of PCV2 sequences that clustered with sequences outside of Ukraine for genotypes **b**, **d**, **f** and **a** (Figure 2). These results suggest that PCV2 genotypes in wild boar and domestic swine in Ukraine are potentially the result of multiple introductions, or exchanges, across borders. Thus, PCV2 should be analyzed in a regional context (a Central/East European group), keeping in mind the possible long-distance transfer of the virus across Eurasia. It is unclear whether genomic surveillance for PCV2 might provide data for mapping swine disease ecology—however, this is a pressing question not only for understanding PCVAD, but also for the spread of other swine diseases, such as ASF, PEDV, and swine influenza that infect both wild boar and domestic swine [1,2,3].

PCV2 genotype **b** is found in domestic pigs in Europe, Asia, and North America, so it is evident that this, along with genotype **d**, is a widely transmitted genotype of the virus [12]. We discovered 12 new genotype **b** infections in Ukraine; four of the infections fall into a cluster, but the others do not (Figure 2). With the paucity of PCV2 genotype **b** samples from Ukraine, it is not possible to build a robust time-demarked phylogeny. Thus, it is difficult to infer whether there was only one or a few ancestral introductions of this PCV2 genotype **b** subclade into Ukraine, or if there were multiple occurrences.

The complete genome we assembled of the Chernihiv-1 isolate (PCV2/Chernihiv-1/2012) within this subclade had 3 SNV in comparison to its closest match from China (PCV2/China FX1102/2012, Accession JX406426.1). However, there were no amino acid variations in the major proteins involved in PCV2 replication.

We also identified PCV2 genotype **d** in the domestic pig samples and in one wild boar sequence (NCM) sampled before 2012. Genotype **d** infections were rare in domestic pigs in the US and Korea, and in wild boars in Korea and Italy, among other places, before 2015 [12,28,31]. We report the first discovery of a PCV2 genotype **f** in Ukraine, that had infected a wild boar in Poltava *oblast*. The prevalence of this genotype is unknown, but it reflects the diversity of PCV2 in swine in Ukraine.

### 4.5. Limitations

This study is limited by the sample size and lack of longitudinal sequence data on PCV2 circulating in Ukraine. There may be hidden genetic diversity, particularly in genotype **b**, that has not been sampled in either wild boar or domestic swine. This may be especially true for domestic pigs, which had few samples, were sampled from a different time, and sampled from one location. Alternately, the PCV2 genotype **b** circulating among wild boar in Ukraine may actually be relatively homogeneous. We did not observe a stark geographical structure in our data. There was an absence of correlation between genetic and geographical distances using the Mantel test (Figure 3), although this result is tempered by a limited sample size. Consistent with this finding, many PCVAD cases in domestic pigs are thought to be of anthropogenic origin in Ukraine, where agricultural practices play a key role in the spread of this disease. It cannot be assumed they are all genotype **b**, as we also found evidence of genotypes **a** and **f** in wild boar in Ukraine, and co-infections in domestic pigs with genotypes **b** and **d**. Thus, a broader biosurveillance and sequencing effort is needed to understand the potential wild boar-to-domestic swine transmission of PCV2 and other swine diseases.

### 4.6. Nanopore’s Co-Infection Accuracy

Nanopore’s low read accuracy may appear to prevent us from detecting co-infections in our study. We adapted nanopore sequencing and bioinformatics methods from previous study of African swine fever and other swine pathogens to PCV2 analysis herein [26,32,53]. However, it has been shown that the Nanopore raw amplicon reads can be mapped to a close reference with blastn algorithm [54]. The percent of reads misassigned by blastn for genotypes that were 5% different was fewer than 1% of reads [54]. All our samples had at least 99% of reads when the major and minor variants were combined. Also, all the minor variants contributed to over 2% of reads, which is higher than the expected 1% of misassigned reads. The steps used to overcome Nanopore’s low error rate can be found in the Supplementary Methods.

### 4.7. Application of MinION Capacity Building for One Health

To understand PCV2 diversity and spread, we conducted ORF2 (Cap) and full genome PCR amplification and sequencing. Sequencing ORF2 (Cap) alone is a reliable phylogenetic marker for PCV2 genotype assignment [41]. We accomplished this by deploying a cost-effective, accurate, long-read nanopore sequencer (Oxford Nanopore Technologies MinION) in veterinary diagnostic and research labs in Ukraine. 

## 5. Conclusions

Porcine circovirus 2 is the etiological agent of a spectrum of viremic and inflammatory diseases in swine (PCVAD), in part attributable to genetic variation of the pathogen. In this study we found that PCV2 genotypes **b**, **d**, **a** and **f** were present in Ukraine between 2012 and 2019. In domestic pig liver samples, we found PCV2 genotypes **b**, **d**, and **b**/**d** co-infections. In wild boars, we found genotypes **b**, **b**/**a** co-infections, and genotype **f** for the first time in Ukraine. Using phylogenetics, we noted an epizootic cluster of PCV2 genotype **b** in both wild and domestic swine, suggesting a possible ecological interaction in Chernihiv, Kharkiv, and Luhansk oblasts in Ukraine. Our study illustrates the utility and the need for expanded genomic epidemiology to map the reservoirs and transmission routes of PCV2 in Ukraine and to understand the ecological context of this swine disease.

## Figures and Tables

**Table 1:** Porcine circovirus 2 detection in hunted wild boar in Ukraine by PCR (2012).

Region in Ukraine (*Oblast*)	Blood Sera Collected	Positive for PCV2 (PCR)	PCV2 Prevalence (%)	Number Selected for Sequencing
Poltava	15	3	20%	2
Sumy	8	5	63%	-
Zaporizhia	13	12	92%	1
Chernihiv	17	5	29%	4
Chernivtsi	5	2	40%	2
Cherkasy	10	1	10%	-
Kherson	1	0	0%	-
Lviv	13	3	23%	-
Volyn	13	1	8%	1
Luhansk	12	2	17%	2
*Total*	107	34	31.8%	12

**Table 2:** PCV2 ORF2 capsid gene (Cap) amplicon sequencing results.

Sample	Host Type	Sequence Yield (Mbp)	Number of Reads	Mean Quality (Q-Score)	Major PCV2 Genotype
Chernihiv 1	Wild Boar	1349.3	170,304	13	b
Chernihiv 2	Wild Boar	558.0	70,235	13.2	b
Chernihiv 3	Wild Boar	568.1	71,070	13.2	b
Chernihiv 4	Wild Boar	383.1	47,930	13.1	b
Chernivtsi 1	Wild Boar	1149.3	144,338	13.1	b
Chernivtsi 2	Wild Boar	1163.6	146,489	13.2	b
Luhansk 1	Wild Boar	NA	NA	NA	NA
Luhansk 2	Wild Boar	365.1	44,527	13.2	b
Poltava 1	Wild Boar	696.9	87,848	13	b
Poltava 2	Wild Boar	605.2	76,231	12.9	f
Volyn	Wild Boar	320.0	39,156	13.1	b
Zaporizhzhia	Wild Boar	121.1	14,941	13.1	b
Kharkiv 1	Domestic pig	3599.6	453,425	14.5	b
Kharkiv 2	Domestic pig	1240.9	156,283	14.6	d
Kharkiv 3	Domestic pig	1828.5	229,888	14.6	b
Kharkiv 4	Domestic pig	560.0	70,490	14.6	d
Kharkiv 5	Domestic pig	5036.4	635,035	14.5	b
Kharkiv 6	Domestic pig	213.9	26,903	14.6	d

**Table 3:** Co-infections of two PCV2 genotypes in swine in Ukraine. Percent and number of reads binned for each minor variant in a co-infection listed.

Sample	% Minor Genotype	No. Reads	Major Genotype	Minor Genotype
Chernivtsi 1 (wild boar)	3.47	5191	b	a
Chernivtsi 2 (wild boar)	3.93	6000	b	a
Karhkiv 4 (domestic pig)	28.41	28356	d	b
Karhkiv 5 (domestic pig)	5.81	39560	b	d
Karhkiv 6 (domestic pig)	25.82	9398	d	b

## Data Availability

PCV2 consensus genomes and partial (Cap) sequences are available in NCBI GenBank, BioProject PRJNA832222 (Porcine circovirus 2 sequences from Ukraine) with its associated Accessions.

## Supplementary Materials

The following are available online at https://www.mdpi.com/article/10.3390/v14050924/s1. Supplementary Methods File. Table S1. Tree Sequence Accession. Table S2. Percent of reads in each sample from the major and minor variant. Table S3. TableS3_PCV2_WildBoar_NVRI_Ukraine_2015 of additional PCV2 sequences from Ukraine previously in NCBI GenBank.
