# Supplementary material for: Genetic Diversity of Porcine Circovirus 2 in Wild Boar and Domestic Pigs in Ukraine
Source: Viruses. 2022 Apr 28;14(5):924. doi: 10.3390/v14050924 (PMC9142977; doi:10.3390/v14050924)
Supplement: Supplementary file 1 [file viruses-14-00924-s001.zip › viruses-1687781-supplementary/SupplementaryFiles/Supplement Table_S2.pdf]

Supplemental Table S2. Percent of reads in each sample from the major and minor variant.

Sample*	% of reads from major variant	%of reads from minor variant	% of reads mapped to major and minor variant
Chernihiv 1	99.96	NA	99.96
Chernihiv 2	99.99	NA	99.99
Chernihiv 3	99.99	NA	99.99
Chernihiv 4	100	NA	100
Chernivtsi 1	96.49	3.47	99.96
Chernivtsi 2	96.03	3.93	99.96
Luhansk 2	100	NA	100
Poltava 1	99.98	NA	99.98
Poltava 2	100	NA	100
Volyn	99.99	NA	99.99
Zaporizhzhia	99.98	NA	99.98
Kharkiv 1	99.99	NA	99.99
Kharkiv 2	99.89	NA	99.89
Kharkiv 3	99.99	NA	99.99
Kharkiv 4	70.62	28.40	99.02
Kharkiv 5	93.80	5.84	99.64
Kharkiv 6	73.90	25.81	99.71
