# Supplementary material for: Genetic Diversity of Porcine Circovirus 2 in Wild Boar and Domestic Pigs in Ukraine
Source: Viruses. 2022 Apr 28;14(5):924. doi: 10.3390/v14050924 (PMC9142977; doi:10.3390/v14050924)
Supplement: Supplementary file 1 [file viruses-14-00924-s001.zip › viruses-1687781-supplementary/SupplementaryFiles/Supplementary Table_S1.pdf]

Supplementary Table S1: Tree Sequence Accessions

MN510433

MN258759

MN258760

MN258761

MN258762

MN482038

MN482040

MN482056

MN735214

MK987069

MN482042

MN482045

MN482046

MN482047

MN482049

MN482050

MN482052

MN482054

MN482058

MN258758

MN735213

MN196673

MN258756

MN258757

MN258755

MN539513

MN539514

MN539515

MN539516

MN539517

MN539518

MN539519

MN539520

MN539521

MN539522

MN539523

MN539524

MN539525

MN539526

MN539527

MN539528

MN539529

MN539530

MN539531

MN539532

MK552324

MN258753

MN258754

MK585076

MN482039

MN482041

MN482051

MN482053

MN482057

MN258752

MN170517

MN170518

MN170519

MN170520

MN170521

MN170522

MN170523

MN170524

MN170525

MN170526

MN170527

MN170528

MN170529

MN170530

MN170531

MN482037

MN482043

MN482044

MN482048

MN482055

MN735212

MN258751

MT104513

MT104512

MN258749

MN735206

MN735205

MK305871

MK305872

MK305875

MK305876

MK305873

MK305874

MK305877

MK305878

MN258748

MK305879

MK305880

MK305881

MK305882

MK305883

MT104511

MN545963

MN735211

MN735210

MH287045

MN735208

MN735209

MN258747

MH922989

MH922990

MN735207

MH323413

MG786932

MG786933

MG786934

MG798696

MK006039

MK990814

MK990815

MK990816

MK599451

MK599452

MK599453

MK599454

MK599455

MK599456

MK599457

MK599458

MK599459

MK599460

MK599461

MK599462

MK599463

MK599464

MH920547

MH920553

MH920557

MH920560

MH920564

MH920569

MH920570

MH920571

MH920575

MH920576

MH920580

MH920581

MH920582

MH920587

MK504381

MK504382

MK504383

MK504400

MK504401

MK504402

MK504403

MK504404

MK504405

MK504406

MK504407

MK504408

MK504409

MK504410

MK504417

MK504418

MK504419

MK504420

MK504422

MK504423

MK604480

MK604481

MK604482

MK604487

MK604489

MK604494

MK604496

MK426837

MK426838

MK426839

MK424116

MK404178

MK404181

MK405698

MK405699

MK405700

MK405701

MK347349

MK347355

MK347358

MK347362

MK347369

MK347373

MK347375

MK347379

MK347380

MK347383

MK347388

MK347389

MK347391

MK347393

MK347398

MK347399

MK347401

MK347405

MK347409

MK347410

MK347412

MH509735

MH509736

MH593263

MH620789

MH645914

MH656967

MH718995

MH094767

MH094768

MH094769

MH094770

MH094771

MH094772

MH094773

MH094774

MH094775

MH094776

MH094777

MH094778

MH094779

MH094780

MH094781

MH094782

MH094783

MH094784

MH094785

MH094786

MH094787

MH094788

MH094789

MH094790

MH094791

MH094792

MH094793

MH094794

MT104514

MN258746

MH414516

MH414517

MH414518

MK281580

MH922991

MH922992

MH922993

MG182445

MH379263

MH379264

MH379265

MG182444

MN258744

MN258745

MN400446

MH553305

MG813265

MH379262

MG940984

MG182443

MG957151

MH211363

MG813261

MG813262

MG813263

MG813264

MG182442

MF802836

MF802838

MN052989

MN052990

MH373560

MH373554

MG182441

MH373553

MF802835

MH373559

MH373557

MH373558

MH373552

MF802837

MH373556

MH373555

MK377493

MK377494

MK377495

MH191378

MF802834

MF169718

MF169719

MF169717

MF169716

MN052988

MF169715

MF169714

MF169713

MK426833

MN052987

MT423827

MK006032

MK006033

MK006034

MK006035

MK006036

MK006037

MK006038

MT376298

MT376299

MT376300

MT376301

MT376304

MT376305

MT376310

MT376311

MT376312

MT376313

MT376320

MT376321

MT376322

MT376323

MT376324

MT376325

MT376334

MT376339

MT376340

MT376341

MT376342

MT376343

MT376344

MT376345

MT376346

MT376347

MT376358

MT376359

MT376360

MT376361

MT376362

MT376367

MT376368

MT376369

MT376370

MT376371

MT376380

MN266483

MK139828

MK139829

MK139830

MK139831

MK139832

MK139833

MK140460

MK140461

MK140462

MK140463

MK140464

MK140465

MK140466

MK140467

MK140468

MK140469

MK140470

MK140471

MK140472

MK140473

MK140474

MK140475

MK140476

MK140477

MK140478

MH920545

MH920546

MH920549

MH920550

MH920551

MH920552

MH920555

MH920556

MH920558

MH920559

MH920561

MH920562

MH920563

MH920567

MH920568

MH920574

MH920577

MH920579

MH920585

MH920586

MH920588

MH920589

MH931449

MK504384

MK504385

MK504386

MK504387

MK504388

MK504396

MK504397

MK504398

MK504399

MK504415

MK504416

MK504421

MK604483

MK604486

MK604491

MK604493

MK604499

MK604502

MK604504

MK604507

MK604515

MK426834

MK426835

MK426836

MK424114

MK424115

MK404177

MK404179

MK404180

MK421973

MK421975

LC383452

LC383454

LC383455

LC383456

MK050980

MK050981

MK050982

MK050983

MK050984

MK050985

MK050986

MK542370

MK542371

MK542372

MK542373

MK542374

MH509732

MH509733

MK015042

MK015043

MK015044

MH151168

MG732798

MG732799

MG732800

MG732801

MG732802

MG732803

MG732804

MG732805

MG732806

MG732807

MG732808

MG732809

MG732810

MG732811

MG732812

MH046838

MH046839

MH046840

MH059556

MH059557

MH059558

MH059559

MH059560

MH059561

MH059562

MH059563

MH059564

MH059565

MH059566

MH059567

MF677844

MF677845

MF589523

MF589524

MF589528

MF589538

MF679546

MF679547

MF679549

MF679550

MF679551

MF679552

MF679553

MF679563

MF679565

MF679566

MF679567

MF679578

MF679591

MF679596

MF679601

MF679602

MF679603

MG715493

MG715494

MG715495

MG715496

MG715497

MG715498

MG715499

LC310737

LC310738

LC310739

LC310740

MK751862

MF326353

MF326354

MF326355

MF326356

MF326357

MF326358

MF326359

MF326360

MF326361

MF326362

MF326363

MF326364

MF326365

MF326366

MF326367

MF326368

MF326369

MF326370

MF326371

MF326372

MF326373

KY659550

KY659553

MF169712

MF169711

MF169710

MF169709

KY425815

MF737370

MF737371

MF737372

MF737373

MF737374

MF737375

MF737376

MF737382

MF737383

MF737384

KY947552

KY947556

KY947560

KY947566

KY947567

KY947570

KY947576

KY947579

KY947580

KY947581

KY659551

KY659554

MF169708

MF169705

MF169706

MF169707

KY126314

KY126313

MF169704

KY947554

KY947568

KY947571

KY947573

KY947575

KY947578

KY659552

MF169702

MF169703

MF169701

MF169700

MF169669

MF169699

MG813259

MG182440

MG182437

MF169698

KY126312

MF169668

MF169697

MF169695

MF169696

MF169694

MF169692

MF169693

MH341481

MF169690

MF169691

MF169688

MF169689

MF169686

MF169687

KY947557

KY947558

KY947563

KY947564

KY947565

KY947577

KY126311

MF169664

MF169665

MF169666

MF169667

MF169684

MF169685

MF169683

MG744311

MG182439

MF169681

MF169682

MF169680

KX845692

MF169663

MF169679

KX814350

KX845693

MN052969

MN052970

KX865094

MF169660

MF169661

MF169662

MF169678

KX814351

KX845694

MG182438

KY425814

MN052966

MN052967

MN052968

MF169677

KX814349

MG807481

MG807482

MG813260

MF169676

KY126317

MF169675

KY985392

KY985393

KY985394

KY985395

KY985396

KY985397

KY985398

KY985399

KY985400

KY985401

KY985402

KY985403

MF169674

KX814352

KX845695

MF169673

KY656087

KY656088

KY656089

KY656090

KY656091

KY656092

KY656101

KY656102

KY656103

KY656104

KY656105

KY656106

KX510054

KX510055

KX510056

KX510057

KX510058

KX510059

KX510060

KX510061

KX510062

KX510063

KX510064

KX510065

KX510066

KX510067

KX510068

KX510069

KX510070

KX510071

KX510072

KX510073

KX510074

KX510075

KX510076

KX510077

KX510078

KX510079

KX510080

KX510081

KX510082

KX510083

KX510084

KX510085

MF169672

KY569375

KY305203

KY305204

KX352161

MF169671

MF169670

KX668489

KX865093

KY655969

KY655970

KY656062

KY656063

KY656086

KY656096

KY656097

KY656098

KY656099

KX098764

KX098765

KX098766

KX098767

KX098768

KX098769

KX098770

KX098771

KX098772

KX098773

KX098774

KX098775

KX098776

KX098777

KX098778

KX098779

KX098780

KX098781

KX098782

KX510050

KX510051

KX510052

KX510053

KX352160

MN052965

KY655959

KY655960

KY655961

KY655962

KY655963

KY655964

KY655965

KY655966

KY655967

KY655968

KY655972

KY655973

KY656057

KY656058

KY656059

KY656060

KY656061

KY656064

KY656076

KY656077

KY656078

KY656079

KY656080

KY656093

KY656100

KX098751

KX098752

KX098753

KX098754

KX098755

KX098756

KX098757

KX098758

KX098759

KX098760

KX098761

KX098762

KX098763

MN052964

MG833033

KY655955

KY655956

KY655958

KY655971

KY656075

KY656085

KX098737

KX098738

KX098739

KX098740

KX098741

KX098742

KX098743

KX098744

KX098745

KX098746

KX098747

KX098748

KX098749

KX098750

MT376295

MT376296

MT376306

MT376314

MT376315

MT376326

MT376348

MT376349

MT376363

MT376364

MT376372

MH890676

MH920544

MH920548

MH920554

MH920565

MH920566

MH920572

MH920573

MH920578

MH920583

MH920584

MK504389

MK504390

MK504391

MK504392

MK504393

MK504394

MK504395

MK504411

MK504412

MK504413

MK504414

MK604479

MK604485

MK604488

MK604490

MK604492

MK604495

MK604498

MK604501

MK604503

MK604505

MK604506

MK604508

MK604509

MK604510

MK604511

MK604513

MK604514

MK424113

LC383453

MK405696

MK405697

MK347351

MK347352

MK347353

MK347354

MK347361

MK347364

MK347371

MK347374

MK347377

MK347381

MK347385

MK347386

MK347387

MK347400

MK035432

MK035433

MH509731

MH509734

MH465400

MH465401

MH465429

MH465430

MH465431

MH465451

MH465468

MH465469

MH465470

MH465471

MH465472

MH465482

MH351269

MH351270

MH351271

MG732815

MG732816

MG732817

MG732818

MG732819

MG732820

MG732821

MG732822

MG732823

MG732824

MG732825

MG732826

MG732827

MG732828

MG732829

MG732830

MG732831

MG732832

MG732833

MG732834

MG732835

MG893891

MG893892

MG893893

MG893894

MG893895

MG893896

MG807610

MG807611

MG807612

MH055401

MH055402

MH055403

MH055404

MH055405

MH055406

MH055407

MH055408

MH055409

MH055410

MH055411

MH055412

MF677842

MF677843

MH496613

MH496614

MH046794

MH046795

MF631805

MF631806

MF631808

MF631809

MF631810

MF631812

MF631814

MF631815

MF631816

MF631817

MF631818

MF631819

MF631820

MF631821

MF631822

MF631823

MF631824

MF631825

MF631826

MF631827

MF631828

MF631829

MG245866

MG245867

MG870195

MF589529

MF589537

MF679554

MF679555

MF679556

MF679557

MF679558

MF679559

MF679560

MF679561

MF679562

MF679569

MF679577

MF679580

MF679582

MF679590

MF679593

MF679594

MF679595

MF679597

KY613025

KY613026

KY613027

KY613028

KY613029

KY613030

KY613031

KY613032

KY652744

KY652745

KY652746

KY652747

KY810319

KY810320

KY810321

KY810322

KY810323

KY810324

KY810325

MG715490

MG715491

MG715492

KY677756

KY677757

MF385005

MF385006

MF385007

MF385008

MF385009

MF385010

MF385011

MF385012

MF385013

KY806067

LC310733

LC310734

LC310735

LC310736

KX844823

KX855982

KX855983

KX855984

KX867818

KX867819

KX867820

KX867821

KY347898

KY347899

KX981602

KX981603

KX981604

KX828215

KX828216

KX828217

KX828218

KX828228

KX828229

KX828230

KX828231

KX828232

KX828233

KX828234

KX828235

KX828236

KX828237

KX828238

KX828239

KX828240

KX828241

KX352159

KX865092

KX098660

KX098661

KX098711

KX098712

KX098713

KX098714

KX098715

KX098716

KX098717

KX098718

KX098719

KX098720

KX098721

KX098722

KX098723

KX098724

KX098725

KX098726

KX098727

KX098728

KX098729

KX098730

KX098731

KX098732

KX098733

KX098734

KX098735

KX098736

KX247804

KX247817

KX247834

KX247837

KX247841

KU557355

MH341495

MH341496

KU557354

MH341488

KX814348

MF737377

MF737378

MF737379

MF737380

MF737381

KY940543

KY656054

KX098696

KX098697

KX098698

KX098699

KX098700

KX098701

KX098702

KX098703

KX098704

KX098705

KX098706

KX098707

KX098708

KX098709

KX098710

KX247803

KX247808

MH341494

MH341487

MH341489

KX814347

KX169317

KX169318

KX169319

KX169320

KX169321

KX169322

KX169323

KX169324

KX169325

KX169326

KX169327

KX169328

KX169329

KX169330

KX169331

KX169332

KX169333

KX169334

KX169335

KX169336

KX169337

KX668490

KX668491

KX098667

KX098668

KX098669

KX098670

KX098671

KX098672

KX098673

KX098674

KX098675

KX098676

KX098677

KX098678

KX098679

KX098681

KX098682

KX098683

KX098684

KX098685

KX098686

KX098687

KX098688

KX098689

KX098690

KX098691

KX098692

KX098693

KX098694

KX098695

KX247816

KX247821

KY126315

KY985391

KY940534

KY940535

KX668492

KX098659

KX098662

KX098663

KX098664

KX098665

KX098666

KX247789

KX247797

KX247825

KX247833

KX247844

KU697285

KU697286

KU697287

KU697288

KU697289

KU697290

KU697291

KU697292

KX169296

KX169297

KX169298

KX169299

KX169300

KX169301

KX169302

KX169303

KX169304

KX169305

KX169306

KX169307

KX169308

KX169309

KX169310

KX169311

KX169312

KX169313

KX169314

KX169315

KX169316

KX352158

KT795290

KT795289

MG182436

KX247812

KU697271

KU697272

KU697273

KU697274

KU697275

KU697276

KU697277

KU697281

KU697282

KU697283

KU697284

KU935610

KX352157

KY985387

KY985388

KY985389

KY985390

MH341497

KY305201

KY305202

MN052977

MN052978

KX247800

KX247811

KU697239

KU697251

KU697252

KU697253

KU697254

KU697255

KU697256

KU697257

KU697258

KU697259

KU697260

KU697261

KU697262

KU697263

KU697264

KU697265

KU697266

KU697267

KU697268

KU697269

KU697270

KU697278

KU697279

KT795288

MN052971

MN052972

MN052973

MN052974

MN052975

MN052976

KX247794

KX247795

KX247796

KX247826

KU697212

KU697213

KU697214

KU697215

KU697216

KU697217

KU697218

KU697219

KU697220

KU697221

KU697222

KU697223

KU697224

KU697225

KU697226

KU697227

KU697228

KU697229

KU697230

KU697231

KU697232

KU697233

KU697234

KU697235

KU697236

KU697237

KU697238

KU697240

KU697241

KU697242

KU697243

KU697244

KU697245

KU697246

KU697247

KU697248

KU697249

KU697250

KU697280

KT284886

KT284887

KU317498

KU317499

KU317497

MG182435

KU317496

MH341498

KU317495

KY305199

KY305200

KU317493

KU317494

KU317491

KU317492

MF169732

KX247790

KX247832

KU697194

KU697195

KU697196

KU697197

KU697198

KU697199

KU697200

KU697201

KU697202

KU697203

KU697204

KU697205

KU697206

KU697207

KU697208

KU697209

KU697210

KU697211

KT284888

KT284889

MH341483

MH341484

MH341486

KU317490

MH341485

KU317488

KU317489

KU317487

KU317485

KU317486

MH341499

MH341491

KU317483

KU317484

KU317482

KU317481

MF169751

MF169760

KX247814

KX247824

KX247838

KU697174

KU697175

KU697176

KU697177

KU697178

KU697179

KU697180

KU697181

KU697182

KU697183

KU697184

KU697185

KU697186

KU697187

KU697188

KU697189

KU697190

KU697191

KU697192

KU697193

KU317480

KU317479

KU317477

KU317478

KU317476

KT804910

KU317475

KU317474

KU697038

KU697039

KU697041

KX831476

KX831477

KX831478

KX831479

KX831480

KX831481

KX831482

KX831483

KU697022

KU697023

KU697040

KU317473

MF169728

MF169736

MF169755

KY656050

KY656051

KY656052

KY656053

KX247813

KX247843

KU697154

KU697155

KU697156

KU697157

KU697158

KU697159

KU697160

KU697161

KU697162

KU697163

KU697164

KU697165

KU697166

KU697167

KU697168

KU697169

KU697170

KU697171

KU697172

KU697173

KR258797

KU697013

KU697015

KU697016

KU697017

KU697018

KU697019

KU697020

KU697021

KU697026

KU697025

KU697028

KU697032

KU697001

KU697029

KU697031

KU697034

KU697042

KU697014

KU697027

KU697033

KU697035

KU697036

KU697037

KU697043

KU697045

KU697008

KU697011

KU697044

KU697048

KU697049

KU697050

KU697051

KU697052

KU697053

KU697012

KU697030

KU697046

KU697024

KU697065

KU697068

KU697069

KU697074

KU697075

KU697076

KU697055

KU697056

KU697057

KU697058

KU697059

KU697047

KU697064

KU697002

KU697007

KU697060

KU697061

KU697062

KU697063

KU697071

KU697073

KU697077

KU697010

KU697081

KU697009

KU697054

KU697005

KU697066

KU697067

KU697082

KU697004

KU697078

KU697080

KU697003

KU697006

KU697070

KU697083

KU697072

KU697079

KY676799

KY646109

MF169731

KY655974

KY656048

KY656049

KY656055

KY656056

KX247784

KX247822

KU697147

KU697148

KU697149

KU697150

KU697151

KU697152

KU697153

KR058359

KX009482

MF169723

MF169726

MF169727

MF169754

KX247807

KX247818

KX247836

KX247840

KU697141

KU697142

KU697143

KU697144

KU697145

KU697146

MN935166

MN935167

MN935168

MN935169

MN935170

MN935171

MN935172

MN935173

MN935174

MT376297

MT376302

MT376307

MT376316

MT376317

MT376318

MT376327

MT376335

MT376336

MT376350

MT376351

MT376352

MT376353

MT376365

MT376373

MT376374

MT376375

MK604484

MK604497

MK604500

MK604512

LC383448

LC383449

LC383450

LC383451

MK347348

MK347350

MK347356

MK347357

MK347359

MK347360

MK347363

MK347365

MK347366

MK347367

MK347368

MK347370

MK347372

MK347376

MK347378

MK347382

MK347384

MK347390

MK347392

MK347394

MK347395

MK347396

MK347397

MK347402

MK347403

MK347404

MK347406

MK347407

MK347408

MK347411

MK347413

MK035434

MH465411

MH465412

MH465413

MH465428

MH465441

MH465442

MH465443

MH465448

MH465465

MH465466

MH465467

MH465481

MH663459

MH756609

MF631830

MF631831

MF631832

MF631833

MF631834

MF589532

MF589541

MF679568

MF679570

MF679571

MF679576

MF679579

MF679583

MF679585

MF679589

MF679592

KY569376

KY569377

KY569378

KY569379

KY569380

KY569381

KY569382

KY569383

KY806062

KY806063

KY806064

KY806065

KY806068

KY806069

MF314309

MF314310

MF314311

MF314312

MF314313

MF314314

MF314315

MF314316

MF314317

MF314318

MF314319

MF314320

MF314321

MF314322

MF314323

MF314324

MF314325

MF314326

MF314327

MF314328

MF314329

MF314330

KX928993

KX928994

KX928995

KX928996

KX928997

KX928998

KX928999

KX929000

KX929001

KX929002

KX929003

KX929004

KX929005

KX929006

KX929007

KX929008

KX929009

KX929010

KX929011

KX929012

KX929013

KX929014

KX929015

KX929016

KX929017

KX929018

KX929019

KX808464

KX808465

KX808466

KX808467

KX808468

KX808469

KX808470

KX808471

KX808472

KX808473

KX808474

KX808475

KX808476

KX808477

LC310724

LC310725

LC310726

LC310727

LC310728

LC310729

LC310730

LC310731

LC310732

KX844824

KX844825

KX826908

KX831475

KX828214

KX828226

KX828227

KX161669

KU960931

KU960933

KU960934

KU960937

KU960939

KU960940

KU960941

KT867802

KT867803

KT867811

KT867823

KT867824

KT867825

KT867826

KT867827

KT867828

KT867829

KT867830

KT867831

KT867832

KT867833

KT867834

KT868032

MH341482

MH341490

MF169725

MF169757

KY656024

KY656025

KY656026

KY656027

KY656028

KY656029

KY656030

KY656031

KY656032

KY656033

KY656034

KY656035

KY656036

KY656047

KX247828

KU697123

KU697124

KU697135

KU697136

KU697137

KU697138

KU697139

KU697140

KX068222

MH341500

KP637021

MH341493

MF169758

KY656017

KY656018

KY656019

KY656020

KY656021

KY656022

KY656023

KY656083

KY656084

KX247823

KX247839

KU697122

KU697127

KU697128

KU697129

KU697130

KU697131

KU697132

KU697133

KU697134

KU311034

KU311035

KT220420

KU311033

KY656008

KY656009

KY656010

KY656011

KY656012

KY656013

KY656014

KY656015

KY656016

KY656045

KY656046

KY656094

KX247801

KU697115

KU697116

KU697117

KU697118

KU697119

KU697120

KU697121

KU697125

KU697126

KP245918

KP245919

KM974911

KU311031

KU311032

KU311030

KU317472

KY940536

KY940537

KY940538

KY940539

KY940540

KY940541

KY940542

KY656004

KY656005

KY656006

KY656007

KY656072

KY656073

KY656074

KX247799

KU697113

KU697114

KP179231

KP112486

KP081553

KT795287

KR058352

KR058353

MF169720

KY655999

KY656000

KY656001

KY656002

KY656003

KY656081

KX247830

KU697105

KU697106

KU697107

KU697108

KU697109

KU697110

KU697111

KP243193

LC008141

KP081551

KR058358

KP112485

KM235959

KM235960

KM235961

KP112484

KP081552

KR052145

KR052146

MN052985

MN052986

MF169729

MF169741

MF169752

MF169753

MF169756

KY655994

KY655995

KY655996

KY655997

KY655998

KY656042

KY656043

KY656044

KX247827

KU756238

KR058355

KR058356

KR058357

KR058354

KU311028

KU311029

KP081550

KX068219

KU311026

KU311027

MH341492

KP081549

KX960942

KX960944

MN052981

MN052982

MN052983

MN052984

MF169721

MF169749

MF169750

KY655989

KY655990

KY655991

KY655992

KY655993

KY656070

KY656071

KY656095

KX247819

KU697099

KU697100

KU697101

KU697102

KU697103

KU697104

KP081546

KP081547

KU311025

KM272212

KX352156

KU311024

KM455976

KU311023

KM035761

KM035762

MF169724

KY655984

KY655985

KY655986

KY655987

KY655988

KY656068

KY656069

KX247791

KU756237

KM035760

KM880085

KM880086

KM880087

KM880088

KM880089

KX068221

KM503044

KU311022

KP081545

KM624038

KU311021

KM624039

KU311020

KX894318

KX894319

MF169730

MF169759

KY940524

KY940525

KY940526

KY940527

KY940528

KY940529

KY655978

KY655979

KY655980

KY655981

KY655982

KY655983

KY656039

KY656040

KY656041

KY656065

KY656066

KY656067

KX247793

KX247802

KU697096

KU697097

KU697098

KU697112

KP081548

KM455974

KM272211

KP081544

KM455975

KY655977

KY656037

KY656038

KY656082

KX247786

KX247815

KX247829

KU697091

KU697092

KU697093

KU697094

KU697095

KX068220

KY305198

KM245558

KJ956689

KJ956690

KJ956691

KJ956692

KM455973

KM455972

MN052980

MF169722

KY655976

KX247783

KU697087

KU697088

KU697089

KU697090

KP231168

KX352155

KJ437506

KM067384

KM067385

MN052979

KY655975

KX247805

KX247831

KU697084

KU697085

KU697086

MT376303

MT376308

MT376309

MT376328

MT376329

MT376330

MT376331

MT376332

MT376333

MT376337

MT376354

MT376355

MT376356

MT376366

MT376376

MT376377

MT376378

MT376379

LC383445

LC383446

LC383447

MH465403

MH465410

MH465427

MH465438

MH465439

MH465440

MH465456

MH465462

MH465463

MH465464

MH465476

MH465477

MH465478

MH465479

MH465480

MH756607

MH756614

MH756616

MF589526

MF589531

MF589533

MF589534

MF589539

MF589540

MF679575

MF679581

MF679584

MF679587

MF679588

MG229680

MG229681

KY806053

KY806054

KY806055

KY806056

KY806057

KY806058

KY806059

KY806060

KY806061

KY806066

KY806071

MF314286

MF314287

MF314288

MF314289

MF314290

MF314291

MF314292

MF314293

MF314294

MF314295

MF314296

MF314297

MF314298

MF314299

MF314300

MF314301

MF314302

MF314303

MF314304

MF314305

MF314306

MF314307

MF314308

KX826907

KT819159

KT819160

KT819161

KT819162

KT819163

KT819164

KT819165

KT819166

KT819167

KT819168

KT819169

KT819170

KY211020

KX298473

KX298474

KX828225

KT336598

KT336599

KT336600

KT336601

KT336604

KT336605

KT336606

KT336607

KT336608

KX161699

KU960929

KU960930

KU960932

KU960936

KT867804

KT867805

KT867814

KT867815

KT867816

KT867817

KT868031

KT868036

KT868419

KP670418

KP670419

KP313251

KP313252

KP313253

KP313254

KP698401

KP698402

KP698403

KP698404

KP698405

KP231173

KR149371

KP231125

KP231126

KP231140

KP081543

KP081555

LC008137

KX247810

KJ139962

KX352154

KP081554

LC008138

KM880080

KM880081

KM880082

KM880083

KM487709

KF742552

KF742553

KY940531

KM191052

KM191056

KM191064

KM191065

KM191087

KM191093

KM191107

KM191109

KM191113

KM191116

KM191121

KM191122

KM191124

KM487708

KP231124

KM880084

KM434195

KU311019

KP231137

KJ867553

KJ867554

KJ867555

KJ867556

KU311018

KP231123

KP081542

KX247806

KM460824

KM191046

KM191059

KM191062

KM191063

KM191106

KM191118

KP081556

KU311017

KU311016

KY947559

KY947562

KY947569

KY947572

KY947574

KY940532

KY940533

KM191051

KM191061

KM191079

KM191080

KM191081

KM191110

KM191114

KM191120

KM116513

KM116514

KM116515

KJ187306

KF926650

KJ133547

KM360053

KX247835

KM191060

KM191090

KM191112

KM191123

KP231128

KP231127

LC008142

LC004754

LC004745

KJ729074

LC004735

LC008136

LC008139

KY940520

KY940521

KY940522

KY940523

KY940530

KX247809

KX247820

KX247842

KM191073

KM191074

KM191078

KM191084

KM191086

KM191089

KM191092

KM191100

KM191101

KM191102

KM191108

KM191115

KM191117

KM191125

KP081541

LC004744

LC004734

KX352153

KM360049

KM360054

KJ729075

LC004733

KX960941

KX960943

KX960945

KX960946

KX960947

KX960948

KX960949

KY947553

KY947555

KY947561

KX247798

KM191055

KM191058

KM191088

KM191091

KM191094

KM191095

KM191096

KM191097

KM191098

KM191103

KM191104

KM191105

LC004751

LC004752

KJ596438

KM360052

KM360056

KM360057

KT026095

KP081540

LC004749

LC004750

KM360055

MF169733

MF169734

MF169735

KX130941

KT369067

KT369068

KT369069

KT369070

KM191028

KM191029

KM191030

KM191033

KM191034

KM191057

KM191069

KM191076

KM191083

KM191119

KF742544

KF742547

KF742549

LC004743

LC008140

MF169746

KX247792

KM191027

KM191031

KM191032

KM191053

KM191054

KM191067

KM191068

KM191070

KM191077

KM191082

KM191085

KM191126

KP081539

KJ729072

KC821781

KC821782

KC821783

KC821784

KC821785

MF169740

MF169747

KY655957

KX247787

KX247788

KU311010

KU311011

KU311012

KU311013

KU311014

KU311015

KM191023

KM191024

KM191025

KM191026

KM191066

KM191071

KM191072

KM191099

LC004741

LC004742

LC004740

KP081538

KM360050

KM360051

KX247785

KM190983

KM190995

KM191020

KM191021

KM191022

KM191111

LC004739

KX009480

KR816332

LC004753

MF169748

KM191018

KM191019

KM191045

KF035062

MN935178

MN935179

MN935180

MN935181

MN935182

MN935183

MT376319

MT376338

MT376357

LC383444

MH465406

MH465425

MH465426

MH465434

MH465435

MH465436

MH465437

MH465445

MH465446

MH465447

MH465461

MH465474

MH465475

MH756608

MH756610

MH756611

MH756612

MH756613

MH756615

MF589525

MF589535

MF679572

MF679573

MF679574

MF679586

MF679598

MG229675

MG229678

MG229679

MG229682

KY440165

KY440166

KY806042

KY806044

KY806047

KY806049

KY806050

KY806051

KY806070

MF314262

MF314263

MF314264

MF314265

MF314266

MF314267

MF314268

MF314269

MF314270

MF314271

MF314272

MF314273

MF314274

MF314275

MF314276

MF314277

MF314278

MF314279

MF314280

MF314281

MF314282

MF314283

MF314284

MF314285

KY388466

KY388467

KY388468

KY388469

KY388470

KY388471

KY388472

KY388473

KY388474

KY388475

KY388476

KY388477

KY388478

KY388479

KT336602

KT336603

KX161681

KX161682

KX161684

KX161685

KU193766

KU193767

KU960935

KU960938

KT359570

KT867794

KT867795

KT867796

KT867806

KT867807

KT867808

KT867809

KT867810

KT867820

KT867821

KT867822

KT867851

KT867855

KT867858

KT867860

KT867862

KT867864

KT867865

KT867867

KT867869

KT867872

KT867874

KT867876

KT867877

KT867879

KT867881

KT867883

KT867884

KT867886

KT867888

KT867891

KT867894

KT867896

KT867900

KT867901

KT867903

KT867905

KT867907

KT867909

KT867911

KT867912

KT867913

KT867914

KT867915

KT867916

KT867917

KT867918

KT867919

KT867920

KT867921

KT867922

KT867923

KT867924

KT867925

KT867926

KT867927

KT867928

KT867929

KT867930

KT867931

KT867932

KT867933

KT867934

KT867935

KT867936

KT867937

KT867938

KT867939

KT867940

KT867941

KT867942

KT867943

KT867944

KT867945

KT867946

KT867952

KT867957

KT867962

KT867966

KT867970

KT868030

KT868035

KT868364

KT868369

KT868436

KT868522

KR559697

KT261750

KP670420

KP670421

KP867046

KP867047

KP867048

KP867049

KP867050

KP245923

KP245924

KP245925

KP245926

KP245927

KP245929

KP698394

KP698395

KP698396

KP698397

KP698398

KP698399

KP698400

KJ511870

KJ511871

KJ511872

KJ511873

KJ511874

KJ511875

KJ511876

KJ511877

KC527542

KP231117

LC004736

MF169738

MF169739

KJ599673

KM191012

KM191013

KM191014

KM191015

KM191016

KM191017

KF035064

KF035065

KJ680369

KF035060

KF524259

KC751546

MF169737

KM191049

KF035059

KF732649

KF035067

LC004747

LC008134

LC008135

LC004746

LC004748

MF169742

MF169743

MF169744

MF169745

KM191009

KM191010

KM191011

KM191047

KF742545

KF742551

KX960933

KX960932

KX960934

KX960935

KX960936

KX960937

KX960938

KX960939

KX960940

KM190999

KM191000

KM191001

KM191002

KM191003

KM191004

KM191005

KM191006

KM191007

KM191008

JX535296

JX535297

JX204386

KP231161

KP231162

KJ680363

KP768486

KP768485

KP768481

KP768483

KP768479

KP768480

KP768482

KP768484

KJ680357

KJ680365

KP768478

KM191048

KC859451

KJ729073

KP768474

KP768475

KP768476

KJ680360

KP768473

KP768477

KP231115

KP231164

KP768472

KC860786

KP768470

KP768469

KP768471

KJ680358

KJ680364

JX294717

KM191050

KP768468

KX009481

KF530836

KF530837

KC823057

KC823055

KC800636

KC823056

KP768467

KC823054

KC823053

KM924364

KM924366

KM924367

KM924369

KF035066

KF035072

KF035073

KP231101

KC702785

KC702786

KC699529

KC699530

KC699531

KJ680368

KP420200

KP420201

KF035068

KF035069

KF035070

KF035071

KJ680346

KC153106

KJ680354

KC800644

KC800638

KJ680367

KC188796

KP420199

KP420202

KP420203

MN935175

MN935176

MN935177

MH465405

MH465423

MH465424

MH465450

MH465453

MH465454

MH465455

MH465473

MH756606

MH756617

MH756618

MH492006

MF679564

MF679599

MF679600

MG229677

KY806034

KY806035

KY806036

KY806037

KY806039

KY806040

KY806043

KY806046

KY806052

MF314227

MF314228

MF314229

MF314230

MF314231

MF314232

MF314233

MF314234

MF314235

MF314236

MF314237

MF314238

MF314239

MF314240

MF314241

MF314242

MF314243

MF314244

MF314245

MF314246

MF314247

MF314248

MF314249

MF314250

MF314251

MF314252

MF314253

MF314254

MF314255

MF314256

MF314257

MF314258

MF314259

MF314260

MF314261

KT719404

KX828213

KX828219

KX828220

KX828221

KX828222

KX828223

KX828224

KX161668

KT867798

KT867800

KT867801

KT867812

KT867813

KT867818

KT867819

KT867850

KT867854

KT867857

KT867951

KT867956

KT867961

KT867965

KT867969

KT867972

KT867973

KT867975

KT867977

KT867992

KT867995

KT867997

KT867998

KT867999

KT868000

KT868001

KT868046

KT868049

KT868052

KT868288

KT868290

KT868373

KT868377

KT868381

KT868384

KT868385

KT868388

KT868390

KT868392

KT868393

KT868394

KT868395

KT868396

KT868397

KT868398

KT868399

KT868400

KT868401

KT868402

KT868403

KT868404

KT868405

KT868406

KT868407

KT868408

KT868409

KT868410

KT868411

KT868412

KT868413

KT868414

KT868415

KT868416

KT868417

KT868435

KT868440

KT216672

KT216673

KT216674

KT216675

KT216676

KT216677

KR559686

KR559689

KR559690

KR559691

KR559692

KR559693

KR559696

KR559706

KM975682

KM975683

KP245920

KP245921

KP245922

KP245928

KC514987

KC514988

KC514989

KC514990

KC514991

KC514992

KC514993

KC514994

KC514995

KC514996

KC514997

KC514998

KC514999

KC515000

KC515001

KC515002

KC515003

KC515004

KC515005

KC515006

KC515007

KC515008

KC515009

KC515010

KC515011

KC515012

KC515013

KC515014

KC515015

KC515016

KC515017

KC515018

KC515019

KC515020

KC515021

KC515022

KC515023

KC515024

KC515025

KC515026

KC515027

KC515028

KC515029

KM190984

KM190985

KM190986

KM190988

KM190989

KM190990

KM190991

KM190992

KM190993

KM190994

KM190996

KM190997

KM190998

KJ679446

KC620505

KC620508

KC620512

KC620513

KC620515

KC620518

KC238429

KC238430

KC238431

KC238432

KC238433

KC238434

KC238435

KC238436

KC238437

KC238438

KF027491

KF027492

KF027493

KF027494

KF027495

KF027496

KF027497

KC753768

KC753769

KC753770

KC753771

KC753772

KC533811

KC684978

JX534236

JX534237

KP231118

KC800646

KJ680370

JX948773

JX948772

KC800645

KJ680341

KJ680344

JX948771

KJ680350

JX948786

KC800639

KC800640

KC800641

KC800642

JX982225

JX948776

JX948784

KP231121

KJ680347

KJ680348

KF035061

KF035063

KJ680351

KJ680353

JX948782

JX948770

JX948780

KC800635

JX948768

JX406423

KM191036

JX406420

KC800637

KC800643

KM191127

KM191128

LC004737

LC004738

JX945577

KP231169

KP231170

KP231171

KP231114

KP231119

KP231122

KP231163

JX912914

JX982222

JX945576

JX945575

JX912915

KP231116

JX948783

KF742541

JN639856

JN639857

JX982224

KJ128271

KP231167

JX948778

KX960918

KX960922

KX960924

KX960925

KX960926

KX960927

KX960928

KX960929

KX960930

KX960931

KJ680340

KJ680352

JX406421

JX406425

JX948779

JX406419

JX982221

JX948777

KF850468

JX948785

JX274295

JN119256

JN119257

JN119255

JX982227

JN615187

KF850467

JX982223

KM042407

JX406426

KF850466

KM042406

JX406422

KM042405

JX948775

KM042404

KM042415

JF928002

JF928003

JF928004

JF928005

JF928006

KC823059

KF850465

KP231158

JX982219

JX982220

KJ680361

KJ680362

JF899334

KM042414

KM191038

KM191039

KM191040

KM191041

JQ413808

JF683389

JN382157

JN382158

JN382159

JN382160

JN382161

JN382162

JN382163

JN382164

JN382165

JN382166

JN382167

JN382168

JN382169

JN382170

JN382171

JN382172

JN382173

JN382174

JN382175

JN382176

JN382177

JN382178

JN382179

JN382180

JN382181

JN382182

JN382183

JN382184

JX948774

KJ128274

KP231156

KP420190

KP420191

KP420193

KP420195

KP420196

KM191042

KJ680359

KJ680366

JN382185

JN382186

JN382187

JN382188

JN382189

JN382190

JN382191

JN382192

KM042413

KF850463

KF850464

KM624033

KP420181

KP420182

KP420183

KP420184

KP420185

KP420186

KP420187

KP420192

KP420194

KM191035

KM191037

KJ136262

LC383443

MH465414

MH465452

MF679548

KY806030

KY806031

KY806032

KY806033

KY806038

KY806041

KY806045

KY806048

MF314220

MF314221

MF314222

MF314223

MF314224

MF314225

MF314226

KX161687

KT868029

KT868034

KT868039

KR559687

KR559695

KR559701

KR559707

KR559708

KR559709

KR559710

KR559711

KR559712

KR559713

KR559714

KR559715

KR559716

KR559717

KR559718

KR559719

KR559720

KR559721

KR559722

KR559723

KR559724

KR559725

KC514965

KC514966

KC514967

KC514968

KC514969

KC514970

KC514971

KC514972

KC514973

KC514974

KC514975

KC514976

KC514977

KC514978

KC514979

KC514980

KC514981

KC514982

KC514983

KC514984

KC514985

KC514986

KM624037

KJ920205

KC620502

KC620503

KC620504

KC620506

KC620507

KC620510

KC620516

KC620517

KC620519

KC620520

KC533812

JQ181585

JQ181586

JQ181587

JQ181588

JQ181589

JQ181590

JQ181591

JQ181592

JQ181593

JQ181594

JQ181595

JQ181596

JQ181597

JQ181598

JQ181599

JQ181600

JQ181601

JQ181602

JQ181603

JQ181604

JQ181605

JQ181606

JQ181607

JQ002671

JQ002672

JF682793

JF682794

JN411095

KP231159

KC336417

KC823058

KC336418

KP231166

KP420197

JF683392

JF683393

JF683394

JF683395

KJ680355

KJ680356

KC924956

JX982228

KF850462

KP420188

KP420189

KP420198

JF718784

JQ866920

KP231157

KP231160

KP231144

KP231146

KF850461

KP231145

KF742540

KF742550

JX678978

KX904946

KP231147

KP231148

HQ378166

KC447454

JF683405

HQ650833

KP231149

JF827599

KX960920

KM624036

KX828581

KX960919

KX904952

JF683406

JF927990

KX960916

KX960921

KX904947

KX904953

KX960923

KX856425

KC800634

KP231150

HQ378163

HM535641

KP231151

JX982226

JX948769

JQ955679

JF272499

HM535639

HM535640

HM755880

HM755881

KF530835

HQ693093

KM042403

KP231100

KF850460

JF683402

JF927984

JF927985

JF927986

JF927987

JF927988

JF927989

KJ680343

HQ378162

KP231152

HQ831540

HQ378167

JF272497

KP231153

JQ866918

JQ866919

HQ378157

HQ378158

KF850459

JX948781

JF683399

MH465399

MF314202

MF314203

MF314204

MF314205

MF314206

MF314207

MF314208

MF314209

MF314210

MF314211

MF314212

MF314213

MF314214

MF314215

MF314216

MF314217

MF314218

MF314219

KX161666

KX161667

KX161676

KX161677

KX161680

KX161688

KX161689

KT867950

KT867955

KT867985

KT867988

KT867991

KR559688

KR559694

KR559702

KR559703

KR559704

KR559705

KJ094599

KJ094600

KJ094601

KJ094602

KJ094603

KJ094604

KJ094605

KJ094606

KC620509

KC620511

KC620514

JX099784

JX099785

JQ806749

JN176181

JF682791

JF682792

JN411098

JF317584

JF317585

JF317586

JF317587

JF317588

JF317589

JN006445

JN006446

JN006448

JN006450

JN006459

JN006460

JN006461

JN006462

JN006463

JN006464

JN006465

HQ738639

HQ738641

HQ693092

KP231141

KP231142

KF850469

KJ680349

JF272498

GU574204

GU938302

HQ378161

KP231143

KM624035

KF742548

JQ866917

GU450329

HQ378160

KF850458

HM003569

HQ113117

HQ113121

HQ113118

HQ113119

HQ113120

JF683408

HM009330

HM003570

GU252369

HQ378159

HM776452

GU001709

GU001710

GU450328

GU938303

GU938304

GU450327

GU370063

GU370064

HQ378165

HQ831538

KX904948

KX904951

KJ128269

KP231154

KP231155

KX904950

KX904945

JF683404

JF683407

HM776442

GQ845028

KJ128270

KJ128273

GU083583

HQ831539

HM776443

HM776444

HM776447

HM776448

GQ845027

HQ378164

KM042402

JQ866916

GU083582

HM776437

HM776438

HM776450

GU017735

GQ996404

GQ915288

GQ915289

GQ845026

KM042401

KC473168

HM030908

HM776441

HM776449

HM776451

GQ845025

KM042400

KM042412

KM042398

KM042399

KM042411

KX904949

HM009331

HM009332

KP231108

KP231113

KP016747

LC383440

LC383441

LC383442

MH465421

MH465422

MH465459

MH465460

KY806022

KY806023

KY806024

KY806025

KY806026

KY806027

KY806028

KY806029

MF314196

MF314197

MF314198

MF314199

MF314200

MF314201

KX161670

KX161671

KX161683

KT867849

KT867853

KT867949

KT867954

KT867960

KT867964

KT867968

KT868285

KT868418

KT868439

KT868443

KT868446

KT868449

KR559698

KR559699

KR559700

KJ679445

JX099780

JX099781

JX099782

JX099783

KC835197

KC249977

JN133305

HQ231328

JN411094

JN411096

JN411097

JN411099

JN411100

JF317565

JF317566

JF317567

JF317568

JF317569

JF317570

JF317571

JF317572

JF317573

JF317574

JF317575

JF317576

JF317577

JF317578

JF317579

JF317580

JF317581

JF317582

JF317583

HQ591378

HQ591379

HQ591380

HQ591381

JN006444

JN006447

HQ738640

GU799576

GQ995581

GQ995582

GQ995583

GQ995584

HM102350

GU325756

GU325757

GU325758

GU325759

GU325760

GU325761

GU325762

GU325763

GU325764

GU325765

GU325766

GU325767

GU325768

GU325769

GU325770

GQ449672

GQ359008

KC473166

GQ359011

HM038028

HM038017

KC907703

JF683401

FJ948167

FJ948168

KJ680345

HM776439

HM776440

HM776453

FJ644927

KP231103

KP231135

GQ359002

GQ359003

GQ359004

GQ359005

GQ359006

GQ359007

KP231105

KP231133

KP231110

KP231111

KM042410

JQ866915

HM038032

KC473167

FJ623185

HQ831537

KP231107

KP231138

KP231139

KM042397

KM042396

KP231104

KP231132

JN989553

JN989554

JN989555

JN989556

JN989557

JQ866914

KP231102

HM038030

KX894321

FJ644930

KP231112

KP231136

HM038034

GU124593

KP231129

KP231131

KM042395

HM038022

HM565925

GQ404807

GQ404808

FJ644932

FJ440338

FJ233905

FJ233906

EU780074

KM042409

EU780073

HM776445

HM776446

FJ644928

FJ644929

FJ644931

KM624032

KF742546

KM042394

KP231099

KP231120

KP231130

KP231172

HQ831519

GU450330

GQ359010

EU656143

HQ831536

KP231134

HM038025

KP231165

KY084478

HM565924

FJ644922

EU921256

EU921257

HQ831522

EU555439

EU684164

HQ831520

HM009334

KJ128272

HQ831534

EU521709

HQ402903

GQ359009

HM565921

FJ594471

EU921254

EU921255

LC383438

LC383439

MH465398

MH465402

MH465404

MH465419

MH465420

MH465444

MH465449

MH465458

KY806000

KY806001

KY806002

KY806003

KY806004

KY806005

KY806006

KY806007

KY806008

KY806009

KY806010

KY806011

KY806012

KY806013

KY806014

KY806015

KY806016

KY806017

KY806018

KY806019

KY806020

KY806021

KX161665

KX161672

KX161678

KX161696

KT867982

KT868028

KT868338

KT868438

KT868445

KT868448

KT868452

KT868455

KT868458

KT868461

KT868465

KT868468

KT868470

KT868473

KT868476

KT868479

KT868482

KT868485

KT868488

KT868491

KT868494

KR054744

KJ415567

KJ415568

JX099786

KC618389

JX512858

KC261600

KC261601

HQ231329

HQ591365

HQ591366

HQ591367

HQ591370

HQ591371

HQ591374

HQ591375

HQ591376

HQ591377

JN006443

JN006449

JN006451

JN006452

JN006453

JN006454

JN006455

JN006456

JN006457

JN006458

FJ804417

GU325753

GU325754

GU325755

EU909686

EU909687

EU909688

FJ870967

FJ870968

FJ870969

FJ870970

FJ870971

FJ870972

FJ870973

FJ870974

FJ870975

FJ870976

FJ598044

FJ598045

KF383132

EU886638

KJ680342

EU886637

HM038033

JF927983

HM565919

AB512125

AB512126

AB512127

AB512128

AB512129

AB512130

AB512131

AB512132

AB512133

AB512134

AB512135

AB512136

AB512137

AB512138

AB512139

AB512140

EU521707

KM624031

EU521708

KP231106

KP231109

HQ831533

FJ384965

HM565920

HM009329

GU252370

GQ359000

KF742542

KF742543

FJ384969

HQ831526

GQ358992

GQ358995

JF683403

HM565922

HQ701665

HQ735207

HM009333

FJ501957

HM038023

FJ384968

FJ384966

FJ384967

FJ041151

HM565923

FJ660967

HQ701666

FJ158602

GQ358998

GQ358999

HM038031

HF542107

JF683397

GQ404799

FJ712215

EU296794

HM038026

GQ358994

GQ227412

AB361577

HM038029

HQ831535

AB361579

AB361567

AB361575

AB361581

AB361573

AB361571

GQ358993

AB361585

FJ660970

FJ660971

AB361569

AB361583

GQ404803

GQ404804

FJ712216

FJ233907

FJ233908

FJ233909

FJ233910

EU750909

FJ660969

HQ831523

KC473165

GQ404805

GQ404806

EU281623

EU545546

EU545543

KM624030

FJ660968

JQ387582

HQ831525

GQ358997

JF683398

GQ404802

FJ426398

HM565918

KM624034

EU735558

EU735559

EU735561

EU735562

EU735563

EU735564

LC433842

LC433843

LC433844

MH465407

MH465408

MH465409

KX161673

KX161674

KX161675

KX161693

KX161694

KX161695

KX161697

KT867835

KT867836

KT867837

KT867848

KT867947

KT867959

KT867984

KT867987

KT867990

KT867994

KT868002

KT868004

KT868006

KT868038

KT868043

KT868048

KT868054

KT868057

KT868059

KT868063

KT868066

KT868068

KT868069

KT868072

KT868073

KT868075

KT868076

KT868078

KT868079

KT868080

KT868082

KT868084

KT868086

KT868088

KT868090

KT868092

KT868094

KT868097

KT868101

KT868102

KT868105

KT868107

KT868109

KT868111

KT868113

KT868114

KT868116

KT868118

KT868120

KT868122

KT868124

KT868126

KT868128

KT868130

KT868132

KT868133

KT868134

KT868136

KT868138

KT868140

KT868143

KT868145

KT868146

KT868149

KT868151

KT868153

KT868155

KT868158

KT868160

KT868162

KT868164

KT868166

KT868168

KT868170

KT868172

KT868174

KT868176

KT868177

KT868179

KT868181

KT868287

KT868289

KT868292

KT868294

KT868313

KT868317

KT868321

KT868323

KT868344

KT868347

KT868363

KT868365

KT868368

KT868372

KT868376

KT868380

KT868387

KT868420

KT868428

KT868429

KT868431

KT868432

KT868442

KT868451

KT868454

KT868457

KT868460

KT868463

KT868464

KT868467

KT868469

KT868472

KT868475

KT868478

KT868481

KT868484

KT868487

KT868490

KT868493

KT868496

KT868498

KT868500

KT868502

KT868505

KT868506

KT868507

KT868508

KT868509

KT868511

KT868512

KT868514

KT868516

KM190987

KC835194

KC835196

JQ994269

HQ591368

HQ591372

HQ591373

JF690911

JF690912

JF690913

JF690914

JF690915

JF690916

JF690917

JF690918

JF690919

JF690920

JF690921

JF690922

JF690923

GU049340

HM641752

HM009338

GU808525

GQ911590

FJ905459

FJ905460

FJ905463

FJ905467

FJ905468

FJ905470

FJ905471

HQ831528

JF683400

EU545547

AB361572

AB361578

AB361580

AB361576

AB361574

AB361570

AB361568

EF421971

EF421972

EF421973

HQ831527

AB361566

EF421967

EF421968

EF421969

EF421970

EU545544

AB361582

AB361584

EF493837

FJ158606

FJ158603

FJ158605

HM038027

HM038021

FJ158604

FJ158607

GQ404798

EU126887

EF493838

EF493839

DQ910865

GU247988

DQ910866

EU545549

KM604667

JF290418

GQ404800

GQ404801

EF493842

EF493840

EF493841

GU247991

FJ218000

FJ218001

FJ218002

FJ388889

LC383437

MH465418

MH465432

MH465433

KX161663

KX161664

KX161679

KX161686

KX161690

KX161691

KX161692

KX161698

KT867797

KT867799

KT867839

KT867841

KT867843

KT867844

KT867845

KT867846

KT867847

KT867852

KT867856

KT867859

KT867861

KT867863

KT867866

KT867868

KT867870

KT867871

KT867873

KT867875

KT867878

KT867880

KT867882

KT867885

KT867887

KT867889

KT867890

KT867892

KT867893

KT867895

KT867897

KT867898

KT867899

KT867902

KT867904

KT867906

KT867908

KT867910

KT867948

KT867953

KT867958

KT867963

KT867967

KT867971

KT867974

KT867976

KT867978

KT867979

KT867980

KT867981

KT867983

KT867986

KT867989

KT867993

KT867996

KT868003

KT868005

KT868007

KT868008

KT868009

KT868010

KT868011

KT868012

KT868013

KT868014

KT868015

KT868016

KT868017

KT868018

KT868019

KT868020

KT868021

KT868022

KT868023

KT868024

KT868025

KT868026

KT868037

KT868041

KT868042

KT868045

KT868047

KT868050

KT868053

KT868056

KT868061

KT868065

KT868067

KT868071

KT868074

KT868077

KT868081

KT868083

KT868085

KT868087

KT868089

KT868091

KT868093

KT868095

KT868096

KT868098

KT868099

KT868100

KT868103

KT868104

KT868106

KT868108

KT868110

KT868112

KT868115

KT868117

KT868119

KT868121

KT868123

KT868125

KT868127

KT868129

KT868131

KT868135

KT868137

KT868139

KT868141

KT868142

KT868144

KT868147

KT868148

KT868150

KT868152

KT868154

KT868156

KT868157

KT868159

KT868161

KT868163

KT868165

KT868167

KT868169

KT868171

KT868173

KT868175

KT868178

KT868180

KT868182

KT868183

KT868184

KT868185

KT868186

KT868187

KT868188

KT868189

KT868190

KT868191

KT868192

KT868193

KT868194

KT868195

KT868196

KT868197

KT868198

KT868199

KT868200

KT868201

KT868202

KT868203

KT868204

KT868205

KT868206

KT868207

KT868208

KT868209

KT868210

KT868211

KT868212

KT868213

KT868214

KT868215

KT868216

KT868217

KT868218

KT868219

KT868220

KT868221

KT868222

KT868223

KT868224

KT868225

KT868226

KT868227

KT868228

KT868229

KT868230

KT868231

KT868232

KT868233

KT868234

KT868235

KT868236

KT868237

KT868238

KT868239

KT868240

KT868241

KT868242

KT868243

KT868244

KT868245

KT868246

KT868247

KT868248

KT868249

KT868250

KT868251

KT868252

KT868253

KT868254

KT868255

KT868256

KT868257

KT868258

KT868259

KT868260

KT868261

KT868262

KT868263

KT868264

KT868265

KT868266

KT868267

KT868268

KT868269

KT868270

KT868271

KT868272

KT868273

KT868274

KT868275

KT868276

KT868277

KT868278

KT868279

KT868280

KT868281

KT868282

KT868283

KT868284

KT868286

KT868291

KT868293

KT868295

KT868296

KT868297

KT868298

KT868299

KT868300

KT868301

KT868302

KT868303

KT868304

KT868305

KT868306

KT868307

KT868308

KT868309

KT868316

KT868320

KT868322

KT868325

KT868327

KT868329

KT868339

KT868340

KT868341

KT868342

KT868343

KT868345

KT868346

KT868348

KT868349

KT868350

KT868351

KT868352

KT868353

KT868354

KT868355

KT868356

KT868357

KT868358

KT868359

KT868360

KT868361

KT868367

KT868371

KT868375

KT868379

KT868383

KT868386

KT868389

KT868391

KT868421

KT868422

KT868423

KT868424

KT868425

KT868426

KT868427

KT868433

KT868434

KT868437

KT868441

KT868444

KT868447

KT868450

KT868453

KT868456

KT868459

KT868462

KT868466

KT868471

KT868474

KT868477

KT868480

KT868483

KT868486

KT868489

KT868492

KT868495

KT868497

KT868499

KT868501

KT868503

KT868504

KT868510

KT868513

KT868515

KT868517

KT868518

KT868519

KT868520

KT868521

KC620544

KC620545

KC620546

KC620547

KC620548

KC620549

KC620550

KC620551

KC620552

JX679498

JQ994268

JQ994270

JQ692110

JQ653449

JQ390467

GU049341

GU049342

HM009335

GQ404852

GQ404853

FJ935780

FJ644920

FJ644921

FJ644923

FJ644924

FJ644925

FJ644926

GU247987

GU247992

HQ831532

JF683396

GU247989

HQ831529

FJ483938

GU247990

HM038024

HM038016

HM038018

HM038019

LC383436

KT867838

KT867840

KT867842

KT868027

KT868033

KT868040

KT868044

KT868055

KT868058

KT868060

KT868062

KT868064

KT868070

KT868312

KT868315

KT868319

KT868324

KT868326

KT868328

KT868330

KT868331

KT868332

KT868333

KT868334

KT868335

KT868336

KT868362

KT868366

KT868370

KT868374

KT868378

KT868382

KC620539

KC620540

KC620541

KC620542

KC620543

KC620555

KC620556

KC835192

KC835193

JX512857

JX512859

JX512860

HQ713495

HQ148879

HM009336

HM009337

FJ905461

FJ905462

FJ905464

FJ905465

FJ905466

FJ905469

FJ998185

FJ644555

FJ644556

FJ644557

FJ644558

FJ644559

FJ644560

FJ644561

FJ644562

FJ644563

EU755371

EU755372

EU755373

EU755374

EU755375

EU755376

EU755377

EU755378

EU755379

EU755380

EU755381

HQ831531

GU233804

KM042408

MF964235

KM604666

HM038020

EU346945

HQ831521

KT868051

KT868310

KT868311

KT868314

KC620531

KC620532

KC620537

KC620538

KC835191

KC835195

JX506730

HQ831530

HQ831524

JF927982

JF683391

JF927981

JF927980

JF683388

MH465457

KT868318

KT868430

KC620530

KC620536

KC620553

KC620554

KC835190

EU408780

JF683390

JF927976

KX960917

JF927979

JF927978

JF683387

JF927977

LC383435

MH465415

MH465416

MH465417

KT868337

KC620529

KC620534

KC620535

KC835189

JN133304

FJ644919

KC620525

KC620526

KC620527

KC620528

KX352445

KC620522

KC620523

KC620524

JX512854

MG739618

MG739619

MG739620

MG739621

MF139058

MF139059

MF139061

MF139063

MF139064

MF139065

MF139069

MF139073

MF139074

MF139076

MF139077

MF139078

MF278777

MF278778

MF278779

KC620521

KC620533

JX512856

KY612598

MF139057

MF139060

MF139062

MF139066

MF139067

MF139070

MF139071

MF139072

MF139075

JX512855

MF139068

MF139056

JX512853

NC_005148

MT951192

MT769274

MT769275

MT769276

MT769277

MT769278

MT769279

MT769280

MT769281

MT769282

MT769283

MT769284

MT769285

MT769286

MT769287

MT769288

MT769289

MT769290

MT769291

MT769292

MT769293

MT769294

MT769295

MT769296

MT769297

MT769298

MT769299

MT769300

MT769301

MT769302

MT769303

MT769304

MT769305

MT769306

MT769307

MN258750

MK545025

MK545026

MK545027

MK545028

MK545029

MK545030

MK545031

MK545032

MK545033

MK545034

MK545035

MK545036

MK545037

MK545038

MK545039

MK545040

MK545041

MK545042

MK545043

MK545044

MK545045

MK545046

MK545047

MK545048

MK545049

MK545050

MK545051

MK545052

MK545053

MK545054

MK545055

MK545056

MK426840

MK005831

MK005832

MK005833

MK005834

MK005835

MK005836

MK005837

MK005838

MK005839

MK005840

MK005841

MK005842

MK005843

MK005844

MK005845

MK005846

MK005847

MK005848

MK005849

MK005850

MK005851

MK005852

MK005853

MK005854

MK005855

MK005856

MK005857

MK005858

MK005859

MK005860

MK005861

MK005862

MK005863

MK005864

MK005865

MK005866

MK005867

MK005868

MK005869

MK005870

MK005871

MK005872

MH481748

MH465483

MH465484

MH465485

MH465486

MH465487

MH465488

MH465489

MH465490

MH465491

MG717465

KX641084

KX641085

KX641086

KX641087

KX641088

KX641089

KX641090

KX641091

KX641092

KX641093

KX641094

KX641095

KX641096

KX641097

KX641098

KX641099

KX641100

KX641101

KX641102

KX641103

KX641104

KX641105

KX641106

KX641107

KX641108

KX641109

KX641110

KX641111

KX641112

KX641113

KX641114

KX641115

KX641116

KX641117

KX641118

KX641119

KX641120

KX641121

KX641122

KX641123

KX641124

KX641125

KX641126

KX641127

KX641128

KX641129

KX641130

KX641131

KX641132

KX641133

KX641134

KX641135

KX641136

KX641137

KX641138

KX641139

KX641140

KX641141

KX641142

KX641143

KX641144

KX641145

KX641146

KX641147

KX641148

KX641149

LC278320

LC278321

LC278322

LC278323

LC278324

LC278325

LC278326

LC278327

LC278328

LC278329

LC278330

LC278331

LC278332

LC278333

LC278334

LC278335

LC278336

LC278337

LC278338

LC278339

LC278340

LC278341

LC278342

LC278343

LC278344

LC278345

LC278346

LC278347

LC278348

LC278349

LC278350

LC278351

LC278352

LC278353

MF589527

MF589530

MF589536

MF589542

MF589543

MF589544

MG229674

MG229676

MG252966

MG252967

MG011720

MG011721

MG011722

KY509295

KY509296

MF616413

MF616414

MF616415

MF616416

MF616417

MF616418

MF616419

MF616420

MF616421

MF616422

MF616423

MF616424

MF616425

MF616426

MF616427

MF616428

MF616429

MF616430

MF616431

MF616432

MF616433

MF616434

MF616435

MF616436

MF616437

MF616438

MF142260

MF142261

MF142262

MF142263

MF142264

MF142265

MF142266

MF142267

MF142268

MF142269

MF142270

MF142271

MF142272

MF142273

MF142274

MF142275

MF142276

KU359269

KU359270

KU359271

KU359272

KU359273

KU359274

KU359275

KU359276

KU359277

KU041848

KU041849

KU041850

KU041851

KU041852

KU041853

KU041854

KU041855

KU041856

KU041857

KU041858

KU041859

KP975430

KP975431

KP975433

KP975434

KP975435

KP975436

KP975437

KP975438

KP975439

KP975440

KP975441

KP975442

KP975443

KP975444

KP975445

KP975446

KP975447

KP975448

KP975449

KP975450

KP975451

KP975452

KR868575

LC034919

LC010925

LC010926

LC010927

LC010928

LC010929

LC010930

LC010931

LC010932

LC010933

LC010934

LC010935

LC010936

LC010937

LC010938

KM460823

LC004732

KJ946351

KJ778679

KJ778680

KJ437192

KF871067

KF871068

JX193799

KF695388

KC688418

KC688419

KC688420

HQ202944

HQ202945

HQ202946

HQ202947

HQ202948

HQ202949

HQ202950

HQ202951

HQ202952

HQ202953

HQ202954

HQ202955

HQ202956

HQ202957

HQ202958

HQ202959

HQ202960

HQ202961

HQ202962

HQ202963

HQ202964

HQ202965

HQ202966

HQ202967

HQ202968

HQ202969

HQ202970

HQ202971

HQ202972

HQ202973

JX519293

JX406424

JQ809462

JQ809463

JQ809464

FR823451

JN644769

JN644770

JN644771

JN660055

GU244506

GU244507

HQ395019

HQ395020

HQ395021

HQ395022

HQ395023

HQ395024

HQ395025

HQ395026

HQ395027

HQ395028

HQ395029

HQ395030

HQ395031

HQ395032

HQ395033

HQ395034

HQ395035

HQ395036

HQ395037

HQ395038

HQ395039

HQ395040

HQ395041

HQ395042

HQ395043

HQ395044

HQ395045

HQ395046

HQ395047

HQ395048

HQ395049

HQ395050

HQ395051

HQ395052

HQ395053

HQ395054

HQ395055

HQ395056

HQ395057

HQ395058

HQ395059

HQ395060

HQ395061

JN662673

JN662674

JN662675

JN662676

JN662677

JN662678

JN662679

JN662680

JN662681

JN662682

JN662683

JN662684

HM623764

HM161710

HM161711

HM142894

HM142895

HM142896

HM142897

HM142898

HM142899

HM142900

HM027579

HM027580

FN398022

FN398023

FN398024

FN398025

FN398026

FN398027

EU747085

FJ447482

AB462382

AB462383

AB462384

AB462385

AB462386

AB462387

AB462388

AB462389

AB462390

AB462391

EF493851

EU257511

EU257512

EU257513

EU257514

EU257515

EU257516

EU057184

EU057185

EU057186

EU057187

EU057188

EU057189

EU302139

EU302140

EU302141

EU647557

EU594437

EU594438

EU594439

EU594440

EU589463

EU589623

EU519223

EU519224

EU518247

AB426905

EU450584

EU450585

EU450586

EU450587

EU450588

EU450589

EU450590

EU450591

EU450592

EU450593

EU450594

EU450595

EU450596

EU450597

EU450598

EU450599

EU450600

EU450601

EU450602

EU450603

EU450604

EU450605

EU450606

EU450607

EU450608

EU450609

EU450610

EU450611

EU450612

EU450613

EU450614

EU450615

EU450616

EU450617

EU450618

EU450619

EU450620

EU450621

EU450622

EU450623

EU450624

EU450625

EU450626

EU450627

EU450628

EU450629

EU450630

EU450631

EU450632

EU450633

EU450634

EU450635

EU450636

EU450637

EU450638

EU450639

EU450640

EU450641

EU450642

EU503031

EU503032

EU503033

EU503034

EU503035

EU503036

EU503037

EU503038

EU503039

EU503040

EU444004

EU444005

EU444006

EU444007

EU444008

EU444009

EU444010

DQ915583

DQ915584

DQ915585

DQ915586

DQ915587

DQ915588

EU418626

EU418627

EU391637

EU350548

EU340257

EU340258

EU366323

EU366324

EU366325

EU366326

EF990644

EF990645

EF990646

EU266592

EU266593

EU266594

EU266595

EU266596

EU266597

EU266598

EU266599

EF371518

EF371519

EF371520

EF371521

EF371522

EF371523

EF371524

EF371525

EF371526

EF371527

EF371528

EF371529

EF371530

EF371531

EF371532

EF371533

EF371534

EF371535

EF371536

EF371537

EF371538

EF371539

EF371540

EF371541

EF371542

EF371544

EF371546

EF371547

EF371548

EF371549

EF371550

EF371551

EU274309

EU274310

EU274311

EU274312

EU148503

EU148504

EU148505

EU148506

EU148507

EF565342

EF565343

EF565344

EF565345

EF565346

EF565347

EF565348

EF565349

EF565350

EF565351

EF565352

EF565353

EF565354

EF565355

EF565356

EF565357

EF565358

EF565359

EF565360

EF565361

EF565362

EF565363

EF565364

EF565365

EF565366

EF565367

EF565368

EU283329

EU186062

EU136711

EU136712

EU136713

EU136714

EU136715

EU136716

EU136717

EU136718

EU136719

EU136720

EF675229

EF675230

EF675231

EF675232

EF675233

EF675234

EF675235

EF675236

EF675237

EF675239

EF675240

EF675241

EF675242

EF675244

EU095020

EF989713

EF394774

EF394775

EF394776

EF394777

EF394778

EF394779

EF619037

EF619970

EF619971

EF592575

EF592576

EF560608

EF560609

EF560610

EF452350

EF452351

EF452352

EF452353

EF452354

EF452355

EF452356

EF452357

EF452358

EF452359

EF452360

EF452361

EF452362

EF452363

EF452364

EF452365

EF452366

EF452367

EF524515

EF524516

EF524517

EF524518

EF524519

EF524520

EF524521

EF524522

EF524523

EF524524

EF524525

EF524526

EF524527

EF524528

EF524529

EF524530

EF524531

EF524532

EF524533

EF524534

EF524535

EF524536

EF524537

EF524538

EF524539

EF524540

EF524541

EF524542

DQ997815

DQ997816

DQ997817

EF515839

EF467928

EF458306

EF210106

EF197986

EF197987

EF184220

EF184221

EF184222

EF184223

EF184224

EF184225

EF184226

EF184227

EF184228

EF190922

EF190923

EF190924

EF190925

EF190926

EF190927

EF190928

EF190929

EF190930

EF190931

EF190932

EF190933

EF190934

EF190935

EF190936

EF190937

EF190938

EF190939

EF190940

EF190941

EF190942

EF190943

EF064149

EF064150

EF067852

EF067853

EF028202

DQ923523

DQ923524

DQ220727

DQ220728

DQ220729

DQ220730

DQ220731

DQ220732

DQ220733

DQ220734

DQ220735

DQ220736

DQ220737

DQ220738

DQ220739

DQ870484

DQ856563

DQ856564

DQ856565

DQ856566

DQ856567

DQ856568

DQ856569

DQ856570

DQ856571

DQ856572

DQ856573

DQ856574

DQ856575

DQ856576

DQ856577

DQ856578

DQ856579

DQ856580

DQ856581

DQ861895

DQ861896

DQ861897

DQ861898

DQ861899

DQ861900

DQ861901

DQ861902

DQ629113

DQ629114

DQ629115

DQ629116

DQ629117

DQ629118

DQ629119

DQ629120

DQ629121

DQ629122

DQ629123

DQ629124

DQ629125

DQ629126

DQ629127

DQ629128

DQ629129

DQ629130

DQ629131

DQ629132

DQ629133

DQ629134

DQ629135

DQ648031

DQ534442

DQ397521

DQ364650

DQ322701

AY874163

AY874164

AY874165

AY874166

AY874167

AY874168

AY874169

DQ233257

DQ218419

DQ218420

DQ218421

AY754016

AY754017

AY754018

AY754019

AY754020

AY754021

AY754022

DQ195679

DQ201641

DQ201642

DQ206444

AM086384

DQ180392

DQ180393

DQ151643

DQ141322

DQ104419

DQ104420

DQ104421

DQ104422

DQ104423

AY969004

DQ017036

AY849938

AY943819

AY916791

AY885225

AY864814

AY484407

AY484408

AY484409

AY484410

AY484411

AY484412

AY484413

AY484414

AY484415

AY484416

AY682990

AY682991

AY682992

AY682993

AY682994

AY682995

AY682996

AY682997

AY732494

AY713470

AY691169

AY691679

AY686762

AY686763

AY686764

AY686765

AY678532

AY672600

AY672601

AY651850

AY641542

AY604430

AY613854

AY613906

AY596822

AY596823

AY578327

AY579893

AY556473

AY556474

AY556475

AY556476

AY556477

AY536755

AY536756

AY510375

AY424401

AY424402

AY424403

AY424404

AY424405

AY391729

AY256455

AY256456

AY256457

AY256458

AY256459

AY256460

AY325495

AY321982

AY321983

AY321984

AY321985

AY321986

AY321987

AY321988

AY321989

AY321990

AY321991

AY321992

AY321993

AY321994

AY321995

AY321996

AY321997

AY321998

AY321999

AY322000

AY322001

AY322002

AY322003

AY322004

AY294310

AY291316

AY291317

AY291318

AY288133

AY288134

AY288135

AY217743

AY188355

AY181945

AY181946

AY181947

AY181948

AY180396

AY180397

AY177626

AY146991

AY146992

AY146993

AY094619

AF544024

AY129154

AY129155

AY122275

AF520783

AY099495

AY099496

AY099497

AY099498

AY099499

AY099500

AF465211

AF454546

AY035820

AF381175

AF381176

AF381177

AF264038

AF264039

AF264040

AF264041

AF264042

AF264043

AF201305

AF201306

AF201307

AF201308

AF201309

AF201310

AF201311
